# Spatio‐Temporal Photoactivation of Cytotoxic Proteins

**DOI:** 10.1002/cbic.202200115

**Published:** 2022-05-06

**Authors:** Raquel Cruz‐Samperio, Robert J. Mart, Louis Y. P. Luk, Yu‐Hsuan Tsai, Arwyn T. Jones, Rudolf K. Allemann

**Affiliations:** ^1^ School of Chemistry Cardiff University Main Building, Park Place Cardiff CF10 3AT U.K.; ^2^ Cardiff School of Pharmacy and Pharmaceutical Sciences Cardiff University Redwood Building King Edwards VII Ave Cardiff CF10 3NB U.K.

**Keywords:** barnase, light-activation, inteins, photocaging, saporin

## Abstract

Protein therapeutics offer exquisite selectivity in targeting cellular processes and behaviors, but are rarely used against non‐cell surface targets due to their poor cellular uptake. While cell‐penetrating peptides can be used to deliver recombinant proteins to the cytosol, it is generally difficult to selectively deliver active proteins to target cells. Here, we report a recombinantly produced, intracellular protein delivery and targeting platform that uses a photocaged intein to regulate the spatio‐temporal activation of protein activity in selected cells upon irradiation with light. The platform was successfully demonstrated for two cytotoxic proteins to selectively kill cancer cells after photoactivation of intein splicing. This platform can generically be applied to any protein whose activity can be disrupted by a fused intein, allowing it to underpin a wide variety of future protein therapeutics.

## Graphical abstract

**Successful photoactivation** of proteins in mammalian cells is reported. We use a photocaged‐intein (shown in yellow and pink respectively) to deactivate the protein of interest (shown in grey) and we deliver this construct to mammalian cells to activate it in vivo using light (active protein shown in red and light in blue). In this work we achieved photocontrol of strong cytotoxic proteins in cancer cells, describing a possible new generation of protein therapeutics.

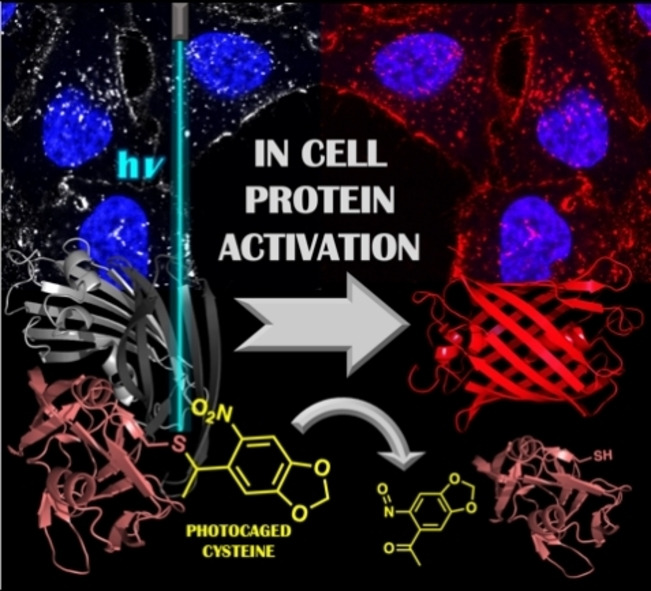

Interest in the use of proteins as therapeutics is rapidly increasing due to their high specificity and biodegradability,^[1]^ however, current protein‐based therapeutics only target extracellular processes or membrane‐bound receptors.^[2]^ The difficulty of delivering proteins across cell membranes severely limits their ability to directly interact with intracellular processes for therapeutic use. Current methods are largely limited to research applications due to either their impracticality in a therapeutic setting (e. g. microinjection, electroporation) or their inability to discriminate between cell types (e. g. unmodified liposomes, nanoparticles, cell‐penetrating peptides).^[3]^ Rather than directing protein delivery to a specific population of cells, here we describe a platform for spatio‐temporal control of therapeutic protein activity by photocaging an intein (Figure 1). Photocages are small aromatic motifs that undergo cleavage upon illumination with light of a particular wavelength.^[4]^ Photocaged amino acids have been used to control protein activity by replacing a functional residue with the corresponding photocaged amino acid chemically, either by solid‐phase peptide synthesis or biologically by genetic code expansion. Where key amino acid residues involved in protein activity are polar, they may be amenable to direct photocaging (e. g., Cys, Lys, Ser, Tyr) to control of protein activity by light,[^5^, ^6^, ^7^] but where photocages cannot be applied, protein activity can alternatively be controlled by splitting the protein sequence into two sections separated by an intein domain.[^8^, ^9^, ^10^] Inteins naturally excise themselves from larger proteins, ligating the flanking extein polypeptides together.

**Figure 1:**
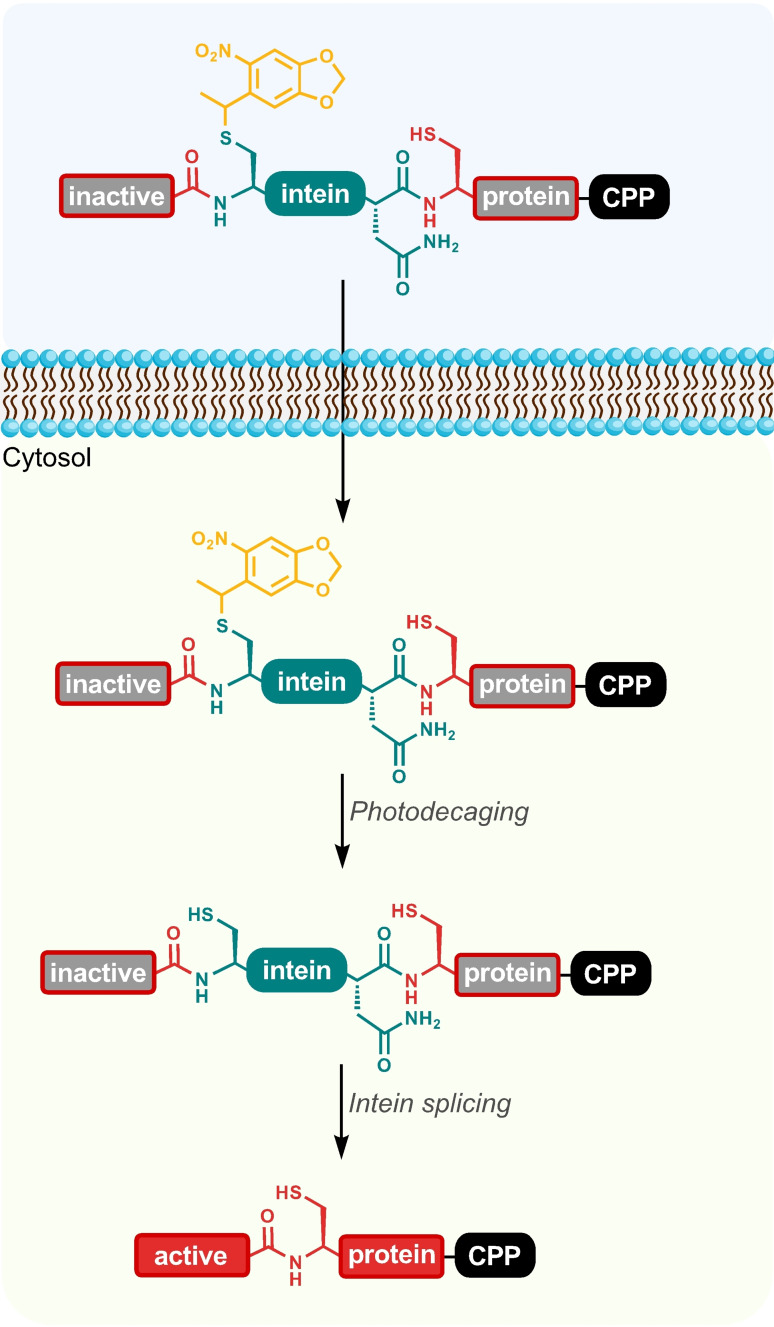
An intein is inserted to perturb the normal protein fold and activity; a photocage (orange) is attached to a cysteine to prevent premature intein activity while a cell‐penetrating peptide (CPP) allows entry into cells.

The trans‐splicing DnaE intein from *Nostoc punctiforme* (*Npu*) is of particular interest due to its tolerance of a wide variety of amino acids in the flanking extein residues and fast splicing rate.[^11^, ^12^, ^13^] *Npu* DnaE intein splicing relies on two cysteine residues at the *N*‐ and *C*‐termini of the split intein domains (Figure S1).[^14^, ^15^] Photocaging one of these key cysteine residues by genetic code expansion[^5^, ^6^, ^8^, ^16^] is known to be sufficient to control intein splicing,[^17^, ^18^] potentially allowing delivery of heterologously expressed photocaged, intein‐inactivated proteins into mammalian cells.

Cell‐penetrating peptides (CPPs) allow protein delivery to the cytosol of mammalian cells and can be readily incorporated into protein therapeutics. Several peptide sequences have been shown promising results during translation into clinical settings.^[19]^ DNA sequences (Figure 2A) were designed encoding cationic CPP sequences derived from the HIV TAT protein, both alone and combined with an endosomolytic peptide sequence (HA2) to improve endosomal escape,^[20]^ and a cyclisation motif to enhance protein uptake.[^21^, ^22^] These sequences were cloned into a plasmid containing DNA encoding *Npu* intein split mCherry with an additional C‐terminal TEV site after the CPP followed by a His_6_‐tag. Expression of these constructs created a functioning intein system that auto‐spliced to give His_6_‐tagged mCherry‐CPP constructs. This ensured CPP modifications allowed correct intein folding and provided model proteins for cell uptake studies. Intein splicing successfully occurred in all four cases (Figure S2), however cell pellets of proteins containing the HA2 sequence showed no color, indicating chromophore maturation had not occurred. These proteins were therefore resuspended and purified in denaturing conditions (Supporting Information). Slow removal of the denaturant yielded protein fractions that became purple with time, indicating successful bypass of a kinetic trap.

**Figure 2:**
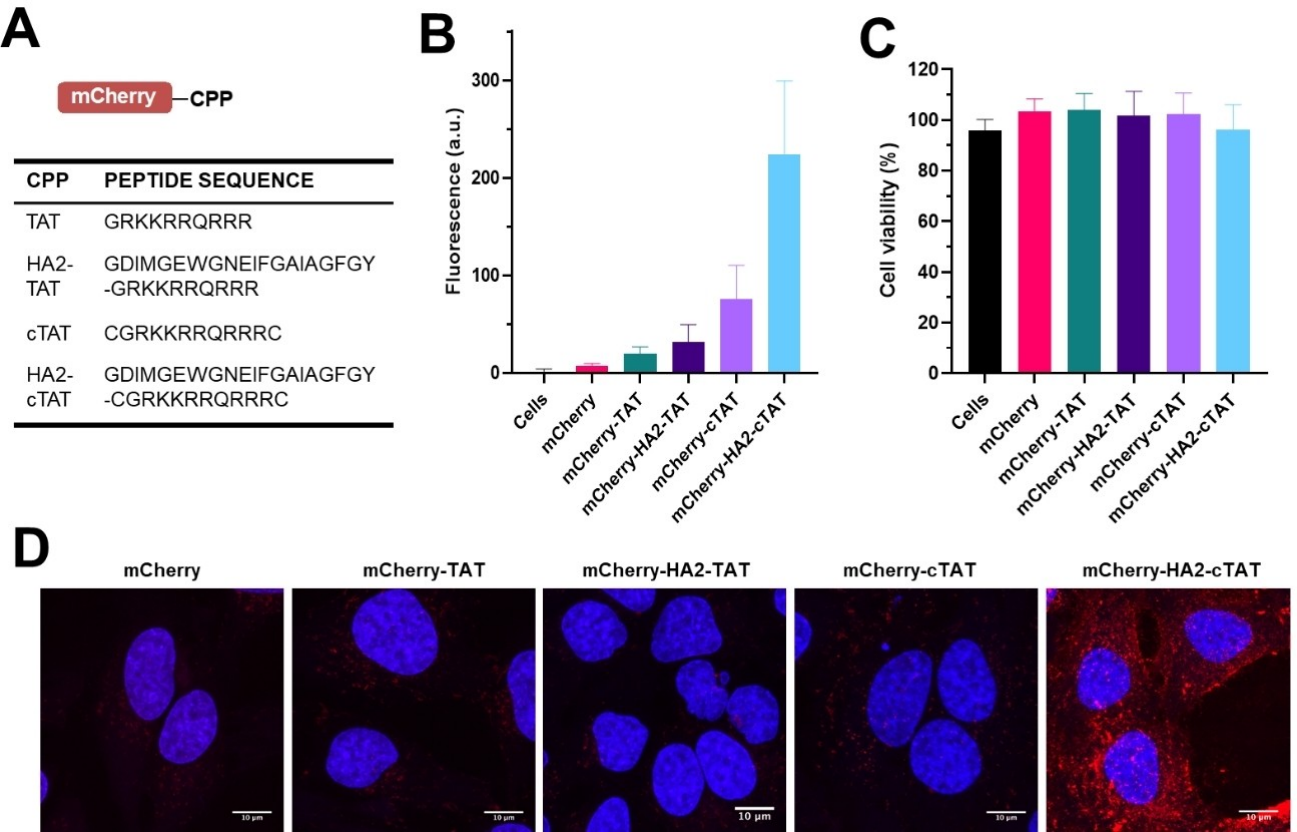
Construction, splicing and cell uptake of intein‐interrupted mCherry‐CPP proteins. (A) Cell penetrating peptide sequences. (B) Flow cytometry of mCherry construct uptake in HeLa cells (values are the man of triplicate measurements of three independent experiments, error bars indicate SD). (C) Viability of HeLa cells treated with mCherry constructs (10 μM) for 2 hours. (D) Confocal microscopy showing the middle section from a z‐stack of images taken from cells incubated with mCherry constructs.

Flow cytometry was used to determine the optimal incubation conditions for HeLa cells (Figure S3) and the relative uptake efficiency of the CPP sequences after 2‐hour incubation with 10 μM mCherry‐CPP (Figure 2B). In agreement with previous reports,[^21^, ^22^] uptake of mCherry with the circular CPP variants was greatly improved over their linear counterparts. Unexpectedly, uptake of mCherry‐HA2‐cTAT was three‐fold greater than mCherry‐cTAT, despite the presence of HA2 not correspondingly improving uptake of the linear mCherry‐HA2‐TAT. To our knowledge, this is the first use of a HA2 and cTAT fusion as a CPP. After observing no CPP related decrease in cell viability in Celltiter Blue assays (Figure 2C), the intracellular distribution of proteins was investigated by confocal microscopy (Figure S4). Analysis of the mid‐line slices of cells (Figure 2D) confirmed the results obtained by flow cytometry and portrayed cytoplasmic mCherry localization. Time lapse movies showed a combination of punctate fluorescence and cytosolic delivery, especially in cells incubated with mCherry‐HA2‐cTAT (Supporting Movies 1–5).

A photocaged intein was then constructed by incorporating *O*‐nitrobenzyl (ONB) photocaged‐intein mCherry (Figure 1) using an engineered PylRS/tRNA_CUA_ pair to charge an O‐nitrobenzyl (ONB)‐protected cysteine in response to an amber (TAG) codon (see Supporting Information).[^5^, ^7^] Non‐photocaged trans‐splicing model systems were used for *in vitro* splicing tests to determine optimal conditions (Figure S5), before testing the photocaged intein activation *in vitro* (Figures S6–8). Intein‐splicing efficiency was determined by comparing fluorescence after mCherry maturation to standards of equal molarity to be less than 5 %, with mass spectrometry (Figure S8) showing the majority of the product to be photodecaged, but unspliced. However, similar yields were obtained using a non‐photocaged trans‐splicing model (Figure S10), suggesting the low yields were due to the flanking extein sequences affecting intein folding. As cellular delivery by CPPs often leads to cargo accumulation in endosomes (∼ pH 4–6), the efficiency of intein splicing at pH values from 3 and 8 (Figure S9) was tested. A significant decrease in fluorescence was observed below pH 6.0, highlighting the importance of achieving endosomal escape (pH 7.2–7.4) to allow efficient intein splicing.

HeLa cells were incubated with photocaged intein‐split mCherry fused to HA2‐cTAT‐CPP for 2 hours, irradiated for 10 minutes with a 360 nm LED lamp, and then incubated at 37 °C for 3 hours to allow time for intein splicing and mCherry chromophore formation. Flow cytometry analysis showed a 3‐fold increase in fluorescence compared to control cells that were not treated with protein (Figure 3A). Light‐activated intein splicing was then applied to saporin, a robust ribosome‐inactivating protein,^[23]^ and barnase, a potent bacterial ribonuclease.^[24]^ Antibody conjugates of both cytotoxins have previously been withdrawn from clinical trials due to strong immune responses caused by the antibodies used to target them to tumors,[^25^, ^26^] but our intein‐based light‐triggered selective activation approach negates the need for antibodies by allowing spatially controlled activation of cytotoxic proteins.

**Figure 3:**
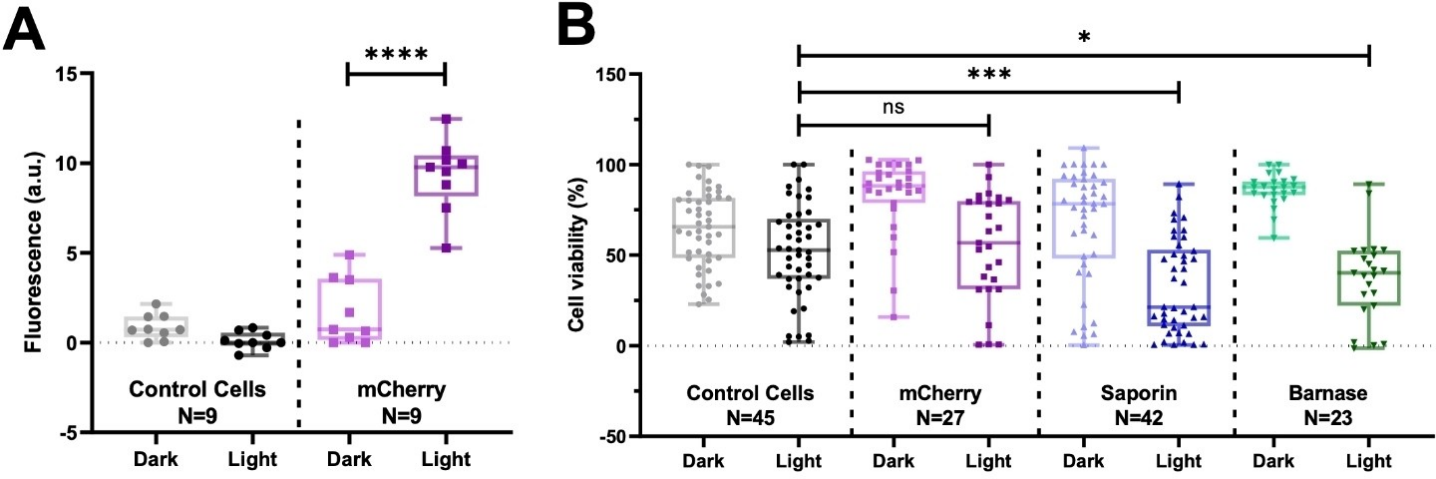
Characterization of delivery system in HeLa cells. (A) Flow cytometry analysis of HeLa cells after light activation of photocaged intein‐split mCherry‐HA2‐cTAT. Box plot shows the mean fluorescence with error bars representing the minimum and maximum value. All individual values are shown in the plot; ***denotes P<0.0001 in unpaired t‐test. (B) Celltiter Blue viability assay of HeLa cells treated with photocaged intein‐split mCherry‐HA2‐cTAT, intein‐split saporin‐HA2‐cTAT, intein‐split barnase‐HA2‐cTAT and control cells. Box plot shows the mean cell viability with error bars representing the minimum and maximum value. All individual values are shown in the plot. ns denotes no significance, * denotes P<0.1, and *** indicates P<0.001 in unpaired t‐test.

Saporin‐S6 is a 253 amino acid protein with two subunits and contains five key catalytic residues, Tyr72, Tyr120, Glu176, Arg179, and Trp208 (Figure S11). The intein was located at Ser171 to divide the main catalytic residues while providing favorable flanking residues for intein splicing. Barnase, consisting of 110 amino acids, contains two key catalytic residues, Glu73 and His102 (Figure S12) and five residues vital for RNA binding and nuclease activity, Lys27, Ser57, Arg59, Arg83 and Arg87.^[27]^ The intein was inserted after Ser38, disrupting a region of the protein at a site which possesses suitable flanking residues. Intein‐split barnase and saporin constructs with HA2‐cTAT CPPs were expressed, refolded, and purified (Supporting Information, Experimental Section). The relative activities of photocaged and photo de‐caged saporin and barnase were investigated by cell viability assays (Figure 3B). Cells were incubated with photocaged intein‐split saporin or barnase and irradiated for 10 minutes using a 365 nm LED UV lamp then incubated overnight at 37 °C to allow intein splicing, nuclease maturation and cytotoxin‐induced apoptosis. Cell viability was strongly affected by the UV irradiation requiring careful optimization of the distance between the wells and the LEDs to ensure reproducibility (Figure S13). Analysis of the viability of irradiated cells (Figure 3B) clearly showed enhanced cell death for populations treated with saporin and barnase compared to control cells. No significant increase in cell death was observed for cells incubated with an mCherry control protein, suggesting we have successfully achieved photoactivation of the cytotoxic proteins using this method. No positive controls for CPP‐conjugated saporin or barnase were produced due to their cytotoxicity to bacteria during expression.

In summary, we demonstrate significant photoactivation of splicing of intein domains rationally inserted into proteins based on existing knowledge of structure and function. Two embodiments of this system incorporating either saporin and barnase allowed selective induction of cell death by light‐activation of intein‐splicing. However, the ultimate potential of this work extends beyond triggering cell death; light‐triggered intein splicing offers the possibility for universal generic de‐ and re‐activation of protein activity, depending on the folding landscape of the protein of interest. Our plug‐and‐play system allows application to a broad range of proteins of interest and any photocage with a cognate tRNA synthetase, including future variants sensitive to longer wavelengths to reduce phototoxicity effects.

## Conflict of interest

The authors declare no conflict of interest.

## Supporting information

As a service to our authors and readers, this journal provides supporting information supplied by the authors. Such materials are peer reviewed and may be re‐organized for online delivery, but are not copy‐edited or typeset. Technical support issues arising from supporting information (other than missing files) should be addressed to the authors.

Supporting Information

Supporting Information

Supporting Information

Supporting Information

Supporting Information

Supporting Information

## Data Availability

The data that support the findings of this study are available in the supplementary material of this article.
