# Supplementary material for: Spatio‐Temporal Photoactivation of Cytotoxic Proteins
Source: Chembiochem. 2022 May 6;23(12):e202200115. doi: 10.1002/cbic.202200115 (PMC9321962; doi:10.1002/cbic.202200115)
Supplement: Supplementary file 1 — Supporting Information [file CBIC-23-0-s003.pdf]

ChemBioChem

Supporting Information

Spatio-Temporal Photoactivation of Cytotoxic Proteins

Raquel Cruz-Samperio, Robert J. Mart, Louis Y. P. Luk, Yu-Hsuan Tsai, Arwyn T. Jones, and Rudolf K. Allemann*

Table of Contents

Materials and Methods
Figures S1 to S23
Tables S1 to S2
Captions for Movies S1 to S5

Experimental Procedures

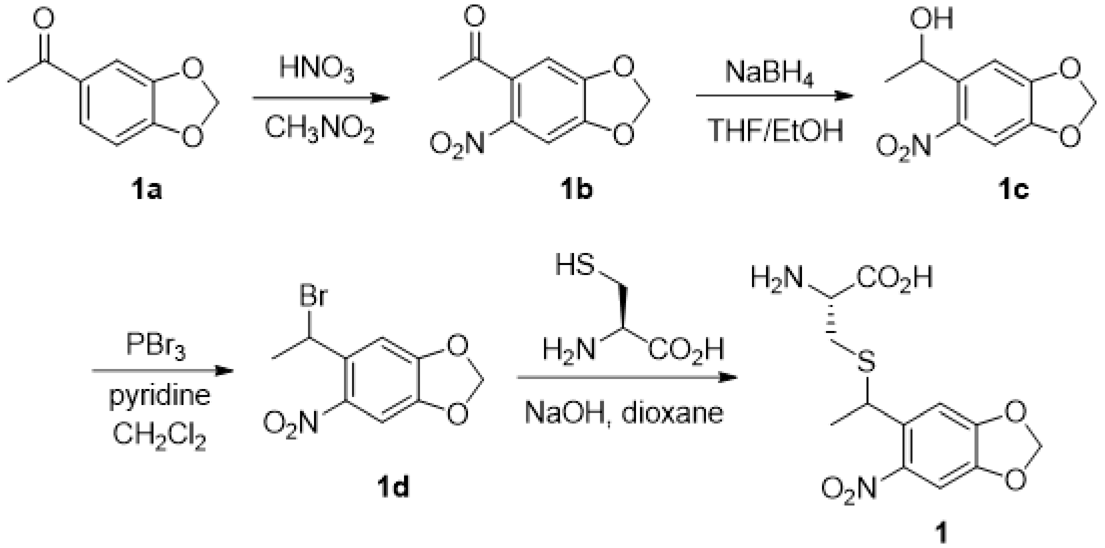

Synthesis of *S*-nitrobenzoylcysteine

Synthesis of 3',4'-(methylendioxy)-6'-nitroacetophenone (**3**). Nitric acid (40 mL, 548.3 mmol, 6.0 eq.) was added dropwise to a stirred solution of 3,4-(methylendioxy)-acetophenone (**2**, 15 g, 91.4 mmol, 1.0 eq.) in nitromethane (170 mL) over 1.5 hours at room temperature. The reaction was then left stirring overnight. The reaction mixture was neutralised with saturated sodium hydrogen carbonate, and the resulting solution extracted with dichloromethane (3 x 50 mL). The combined organic layers were dried over magnesium sulfate, filtered and the solvent was removed under reduced pressure. The product was purified by silica gel chromatography (4:1 hexane/ethyl acetate) to yield 3 as a yellow solid (9.2 g, 48% yield). The product displayed spectroscopic data consistent with those reported previously.1

^{1}H NMR (300 MHz, $CDCl_3$) δ 2.49 (3H, s, -C(=O)C<u>H</u>$_3$), 6.18 (2H, s, -OC<u>H</u>$_2$O-), 6.75 (1H, s, -C<u>H</u>C(NO$_2$)-), 7.55 (1H, s, -C<u>H</u>C(C(=O)CH$_3$)-). **^{13}C NMR** (75 MHz, $CDCl_3$) δ 30.4 (-C(=O)<u>C</u>H$_3$), 103.8 (-<u>C</u>H$_2$O$_2$-), 105.0 (-<u>C</u>HC(NO$_2$)-), 106.4 (-<u>C</u>HC(C(O)CH$_3$)-), 135.3 (-<u>C</u>(C(=O)CH$_3$), 140.3 (-CH<u>C</u>(NO$_2$)-), 149.0 (-<u>C</u>(=O)CHC(NO$_2$)-), 152.8 (-<u>C</u>(=O)CHC(C(=O)CH$_3$)-), 199.3 (-<u>C</u>(=O)CH$_3$). **LRMS** (EI +ve) *m*/*z* 69.00 (33%), 83.95 (54%), 85.95 (35%), 119.01 (33%), 120.02 (73%), 130.99 (24%), 167.02 (45%), 194.01 (100%) [M-CH$_3$]$^+$, 209.03 (66%) [M]$^+$.

Synthesis of methylnitropiperonyl alcohol (**4**). Sodium borohydride (1.5 g, 40.0 mmol, 1.0 eq.) was added portionwise to a solution of 3',4'-(methylendioxy)-6'-nitroacetophenone (**3**, 8.5 g, 40.0 mmol, 1.0 eq.) in tetrahydrofuran (140 mL) and ethanol (140 mL). The reaction was stirred overnight at room temperature. The solution was neutralised with hydrochloric acid solution (1 M) and the solvent was removed under reduced pressure. The resulting solid was partitioned between water and dichloromethane. The aqueous phase was extracted with further dichloromethane (2 x 50 mL) and the combined organic layers were dried over magnesium sulfate, filtered and the solvent removed under reduced pressure to yield **4** as a yellow solid (7.27 g, 85% yield). The product displayed spectroscopic data consistent with those reported previously.[2]

^{1}H NMR (300 MHz, $CDCl_3$) δ 1.54 (3H, d, ^3J = 6.0 Hz, -CH(OH)C<u>H</u>$_3$), 5.46 (1H, q, ^3J = 6.0 Hz, -C<u>H</u>(OH)CH$_3$), 6.11 (2H, s, -C<u>H</u>$_2$O$_2$-), 7.27 (1H, s, -C<u>H</u>C(NO$_2$)-), 7.46 (1H, s, -C<u>H</u>C(CH(OH)CH$_3$)-). **^{13}C NMR** (75 MHz, $CDCl_3$) δ 24.7 (-CH(OH)<u>C</u>H$_3$), 66.2 (-<u>C</u>H(OH)CH$_3$), 103.5 (-<u>C</u>H$_2$O$_2$-), 105.7 (-<u>C</u>HC(NO$_2$)-), 106.9 (-<u>C</u>HC(CH(OH)CH$_3$)-), 139.5 (-<u>C</u>(CH(OH)CH$_3$), 142.1 (-CH<u>C</u>(NO$_2$)-), 147.5 (-<u>C</u>(O)CH<u>C</u>(NO$_2$)-), 153.0 (-<u>C</u>(O)CHC(CH(OH)CH$_3$)-). **LRMS** (EI +ve) *m*/*z* 83.95 (45%), 85.95 (29%), 120.02 (33%), 121.03 (26%), 136.04 (27%), 148.02 (100%), 164.03 (52%), 178.02 (23%), 179.06 (30%), 193.04 (44%), 211.05 (26%) [M]$^+$.

Synthesis of (*R*,*S*)-1-Bromo-1-[4',5'-(methylendioxy)-2'-nitrophenyl]ethane (**5**). An oven-dried 3-necked round-bottom flask containing methylnitropiperonyl alcohol (**4**, 6.5 g, 30.8 mmol, 1.0 eq.) was dried under vacuum for 15 minutes, then purged with argon

for 15 minutes. This cycle was repeated three times. The dried solid was dissolved in dry dichloromethane (150 mL) and the solution cooled to 0 ºC for 30 minutes. Phosphorous tribromide (1.16 mL, 12.3 mmol, 0.4 eq.) was added dropwise over 10 minutes maintaining the temperature of the solution at 0 ºC. Pyridine (0.2 mL, 2.5 mmol, 0.08 eq.) was then added to the reaction mixture. The solution was stirred at 0 ºC for 15 minutes and then at room temperature for 1.5 hours. The reaction was then cooled to 0 ºC again and methanol (7 mL) was added. The solution was stirred at room temperature for 30 minutes. The solvent was then removed under reduced pressure and the residue was partitioned between dichloromethane and saturated sodium bicarbonate solution. The aqueous phase was extracted with further dichloromethane (2 x 50 mL) and the combined organic layers were dried over magnesium sulfate, filtered and the solvent was removed under reduced pressure. The product was purified by column chromatography (1:1 hexane:dichloromethane) to yield **5** as a yellow solid (5.62 g, 67% yield). The product displayed spectroscopic data consistent with those reported previously.[2]

^{1}H NMR (300 MHz, $CDCl_3$) δ 2.03 (3H, d, ^3J = 6.0 Hz, $-CH(Br)C\underline{H}_3$), 5.89 (1H, q, ^3J = 6.0 Hz, $-C\underline{H}(Br)CH_3$), 6.12 (2H, s, $-C\underline{H}_2O_2-$), 7.26 (1H, s, $-C\underline{H}C(NO_2)-$), 7.34 (1H, s, $-C\underline{H}C(C(O)CH_3)-$). **^{13}C NMR** (75 MHz, $CDCl_3$) δ 28.2 ($-CH(Br)\underline{C}H_3$), 43.4 ($-\underline{C}H(Br)CH_3$), 103.8 ($-\underline{C}H_2O_2-$), 105.5 ($-\underline{C}HC(NO_2)-$), 109.3 ($-\underline{C}HC(CH(Br)CH_3)-$), 135.4 ($-\underline{C}(CH(Br)CH_3)$, 148.2 ($-\underline{C}(O)CHC(NO_2)-$), 152.5 ($-\underline{C}(O)CHC(CH(Br)CH_3)-$). **LRMS** (EI +ve) *m/z* 63.02 (26%), 89.04 (41%), 122.03 (25%), 136.04 (39%), 147.04 (27%), 148.03 (87%), 149.03 (42%), 152.03 (57%), 163.06 (28%), 164.03 (85%), 177.04 (33%), 179.05 (65%), 194.02 (100%) $[M-Br]^+$, 272.96 (33%) $[M]^+$, 274.96 (30%) $[M]^+$.

Synthesis of *S*-(1-(6-nitrobenzo[d][1,3]dioxol-5-yl)ethyl)-*L*-cysteine (**1**). A solution of (*R,S*)-1-bromo-1-[4',5'-(methylendioxy)-2'-nitrophenyl]ethane (**5**, 5.0 g, 18.2 mmol, 1.1 eq.) in degassed dioxane (20 mL) was added dropwise to a degassed solution of *L*-cysteine (2.0 g, 16.5 mmol, 1.0 eq.) in sodium hydroxide (33 mL, 0.5 M) over 1 hour. The reaction mixture was stirred at room temperature overnight. Salts formed during the reaction were filtered off and the solution was acidified with hydrobromic acid until precipitation of the product was observed. The product was filtered off to yield **1** as a light yellow solid (2.2 g, 40% yield). The product displayed spectroscopic data consistent with those previously reported.[2]

^{1}H NMR (400 MHz, $NaOD/D_2O$) δ 1.54 (3H, d, ^3J = 6.9 Hz, $-CH(S)C\underline{H}_3$), 2.61-2.75 (2H, m, $-CHC\underline{H}_2S-$), 3.27 (1H, d, ^3J = 5.3 Hz, $-C\underline{H}(S)CH_3$), 4.71 -4.75 (1H, m, $-C\underline{H}CH_2S-$), 6.12 (2H, s, $-C\underline{H}_2O_2-$), 7.27 (1H, s, $-C\underline{H}C(NO_2)-$), 7.37 (1H, d, $-C\underline{H}C(C(O)CH_3)-$). **^{13}C NMR** δ (125 MHz, $NaOD/D_2O$) δ 21.0 ($-CH(S)\underline{C}H3$), 36.9 ($-CH\underline{C}H_2S-$), 39.0 ($-\underline{C}HCH_2S-$), 55.3 ($-\underline{C}H(S)CH_3$), 103.7 ($-\underline{C}H_2O_2-$), 105.2 ($-\underline{C}HC(NO_2)-$), 108.1 ($-\underline{C}HC(CH(Br)CH_3)-$), 136.1 ($-\underline{C}(CH(S)CH_3)$, 142.5 ($-CH\underline{C}(NO_2)-$), 146.8 ($-\underline{C}(O)CHC(NO_2)-$), 152.5 ($-\underline{C}(O)CHC(CH(S)CH_3)-$), 180.9 ($-\underline{C}OOH$). **LRMS** (ES -ve) *m/z* 313.06 (100%) $[M-H]^-$, 314.06 (25%), 367.07 (72%), 421.08 (50%). **HRMS** (ES - ve) calculated 313.0494 for $C_{12}H_{13}N_2O_6S$ $[M-H]^-$, found 313.0493.

Plasmid construction

Amino acid sequences for intein-split mCherry, PCC**2**RS synthetase and intein-split saporin-S6 are detailed in **Table S1**. The pET30b(+) expression plasmid for (144TAG)-mCh-intein-erry with C-terminal His_6 affinity tag, codon-optimised for expression in *Escherichia coli*, was purchased from AddGene. A TAG144C mutant was generated by site-directed mutagenesis using the primers defined in **Table S2**. Cell-penetrating peptide (CPP) sequences were cloned into both plasmids by PCR using the primers described in **Table S2**. CPP sequences were ligated to the plasmid by Gibson assembly for -HA2-TAT or Golden Gate assembly for -TAT, -cTAT and -HA2-cTAT.

Trans-splicing constructs were generated by PCR amplification from the SDM generated plasmid containing (144Cys)-mCh-intein-erry using the primers described in **Table S2**, which attached complementary *Bsa*I sites to expression vectors pETM11 (C-terminal construct) and pET19 (N-terminal) for Golden Gate assembly. Both vectors encode for a N-terminal His_{x6} affinity tag, and pETM11 contained a TEV (*Tobacco Etch Virus*) cleavage site after the affinity tag.

The pUC plasmid of the orthogonal tRNA synthetase, PCC**2**RS, was kindly provided by Dr. Yu-Hsuan Tsai (Cardiff University, UK). Three copies of PCC**2**RS were amplified by PCR using the primers found in **Table S2**, which attached four different complementary *Bsa*I restriction sites for Golden Gate assembly to pCDF expression vector pCDF.

DNA sequences encoding saporin-S6 and barnase, flanked by *Bsa*I sites in 3' and 5' ends, codon-optimised for expression in *E. coli*, were purchased from Thermofisher GeneArt™ as shown in **Table S1**. Products were independently ligated to the pET30b(+) expression plasmid with the -HA2-cTAT CPP sequence by Golden Gate assembly.

PCR amplifications were performed variously with *Pfu* polymerase (New England BioLabs UK Ltd) or PrimeSTAR (Takara Bio Europe). PCR products were purified by agarose gel and ligated by Gibson assembly (Gibson assembly mix from New England BioLabs UK Ltd) or Golden Gate assembly (Using *Bsa*I and T4 ligase from New England BioLabs UK Ltd). All gene sequences were confirmed by sequencing from the T7 promoter and/or the T7 termination sequence (Eurofins Scientific, UK).

Protein expression and purification

mCherry-CPP constructs. Proteins were expressed using BL21 (DE3) *E. coli* (Merck) cells transformed with the plasmid coding for the appropriate (144Cys)-mCherry-CPP constructs grown in LB media supplemented with kanamycin (50 µg/ml). Cultures were grown at 37 ºC until optical density at 600 nm (OD_{600}) equaled or exceeded 0.6, then expression was induced with isopentenyl thiogalactose (0.5 mM, IPTG, Melford) and the cultures were incubated at 25 ºC overnight. Cells were harvested by centrifugation and cell pellets were stored at -20 ºC until required.

mCherry, mCherry-TAT and mCherry-cTAT were purified by suspending the cell pellets in lysis buffer (20 mM Tris, 30 mM NaCl, 20 mM imidazole, pH 8.0) supplemented with egg hen white lysozyme (1 mg/mL, Sigma Aldrich) and phenylmethylsulfonyl fluoride (1 mM, PMSF, Melford). Cells were lysed by sonication (5 s on, 10 s off for a total of 5 min active time) and centrifuged (38,000 x g for 45 min at 4 ºC) to remove cell debris. The supernatant was loaded onto a Ni-NTA column and a step-wise gradient of imidazole was applied (20, 30, 60, 100, 300, 500 mM). Proteins were eluted mostly pure in 60-300 mM imidazole fractions. Imidazole was removed by dialysis against lysis buffer. TEV-His_6 protease and dithiothreitol (DTT) to a final concentration of 5 mM were added to the CPP constructs to remove the C-terminal His_6 tag (overnight digestion at 4 ºC). The solution was passed through a Ni-NTA column to remove the protease and the desired protein was collected in the flow-through. Samples were concentrated to 10 µM (concentration determined by absorbance, ε = 72,000 $M^{-1}\cdot cm^{-1}$) by centrifugal filtration and buffer-exchanged in DMEM (Dulbecco's Modified Eagle Medium, Fisher Scientific) media for the cell assays.

mCherry-HA2-TAT and mCherry-HA2-cTAT were purified by suspending the cell pellets in denaturing lysis buffer (20 mM Tris, 8 M urea, 30 mM NaCl, 20 mM imidazole, pH 8.0) supplemented with PMSF (1 mM). Cells were lysed by sonication (5 s on, 10 s off for a total active time of 5 min) and centrifuged (38,000 x g for 45 min at 4 ºC) to remove cell debris. Supernatant was loaded onto a Ni-NTA column and a step-wise gradient of imidazole was applied (20, 30, 60, 100, 300, 500 mM). Protein was eluted mostly pure in 60-300 mM imidazole fractions. Proteins were refolded by removing urea and imidazole by dialysis against lysis buffer. TEV-His_6 protease and DTT (dithiothreitol) were added to a final concentration of 5 mM to remove the C-terminal His_{x6} tag (overnight digestion at 4 ºC). The solution was loaded onto a Ni-NTA column and the flow-through containing the protein was collected. Samples were concentrated to 10 µM (concentration determined by absorbance, ε = 72,000 $M^{-1}\cdot cm^{-1}$) by centrifugal filtration and buffer-exchanged in DMEM (Dulbecco's Modified Eagle Medium, Fisher Scientific) media for the cell assays. Protein constructs were filter-sterilised with 0.22 µm Millex syringe filter into sterile microcentrifuge tubes, and tubes were stored at -80 ºC until required.

***Trans*-splicing constructs.** Cells were transformed with the appropriate plasmid for the *trans*-splicing constructs and grown in LB media supplemented with ampicillin (100 µg/ml) for the N-terminal construct or kanamycin (50 µg/ml) for the C-terminal construct. Cultures were grown at 37 ºC until OD_{600} = 0.6, then expression was induced with IPTG (0.5 mM) and the cultures incubated at 25 ºC overnight. Cells were harvested by centrifugation and cell pellets were stored at -20 ºC until required.

Both constructs were purified by suspending the cell pellets in denaturing lysis buffer supplemented with PMSF (1 mM). Cells were lysed by sonication (5 s on, 10 s off for 5 min total active time) and centrifuged (38,000 x g for 45 min at 4 ºC). The supernatant was loaded in Ni-NTA column and a step-wise gradient of imidazole was applied (20, 30, 60, 100, 300, 500 mM). Protein was eluted mostly pure in 60-300 mM imidazole fractions. Constructs were refolded, and imidazole was removed, by step-wise dialysis against lysis buffer with decreasing concentrations of urea (4 M urea, 2 M urea, no urea). TEV-His_6 protease and DTT (dithiothreitol) to a final concentration of 5 mM was added to C-terminal construct remove the N-terminal His_6 tag and incubated overnight at 4 ºC. Samples were loaded to a Ni-NTA column and the flow-through containing the protein was collected. Samples were concentrated to 50 µM (concentration determined by Bradford assay) by centrifugation and buffer-exchanged into splicing buffer (20 mM Tris, 300 mM NaCl, 2 mM EDTA, pH 7.3) for kinetic assays.

Splicing kinetics assays

Irradiation studies. (ONB)-mCh-intein-erry (5 µM, in splicing buffer) was irradiated in a quartz cuvette (1 mm path length) by 365 nm light from a UVP Benchtop 2UV transilluminator (~4 mW/cm^2) for specified times. The contents of the cuvette were transferred to black 24-well plates for measuring fluorescence.

Fluorescence spectroscopy by plate reader. Samples were transferred to black 24-well plates and mCherry fluorescence was measured using a VICTOR X5 Multilabel Plate Reader (PerkinElmer) using a 579/25 excitation filter and a 615/8.5 emission filter. Data analysis was performed in Microsoft Excel. Concentrations of mature mCherry were calculated from a calibration curve performed at know concentrations of mCherry determined by absorbance.
Main Text Paragraph.

Results and Discussion

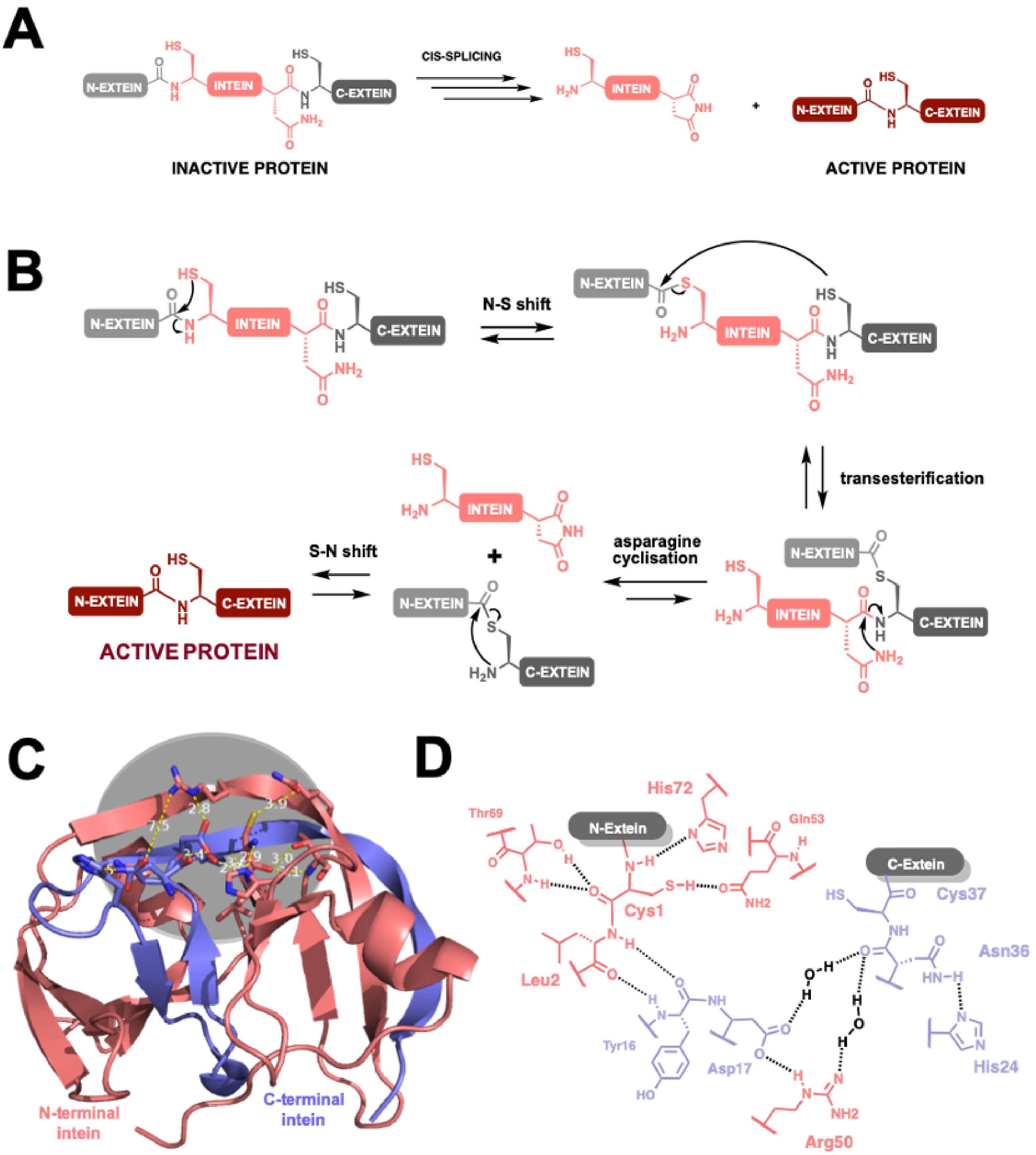

Figure S1. *Npu* DnaE intein: mechanism and structure. (A) General scheme of *cis*-splicing, intein splices itself out of the protein (termed exteins), recovering the protein its native activity. (B) Mechanism of *cis*-intein splicing: (1) N-S shift between terminal residue of N-extein and first residue of intein. (2) Nucleophilic attack of terminal serine to terminal residue of N-extein. Cleavage of intein from N-extein. (3) Internal cyclisation of terminal asparagine. Intein excision from protein. (4) N-S shift to yield fully functional protein. (C) Crystal structure of fused *Nostoc punctiforme* (*Npu*) DnaE. N-terminal domain shown in pink, C-terminal domain in purple (PDB: 4LX3). (D) Catalytic site of *Npu* DnaE. Residues directly involved in the splicing are Cys1 in the N-terminal domain and Asp36 and Cys37 of the C-terminal domain. A web of H-bonds in catalytic site is the driving force for adopting the correct conformation for splicing.

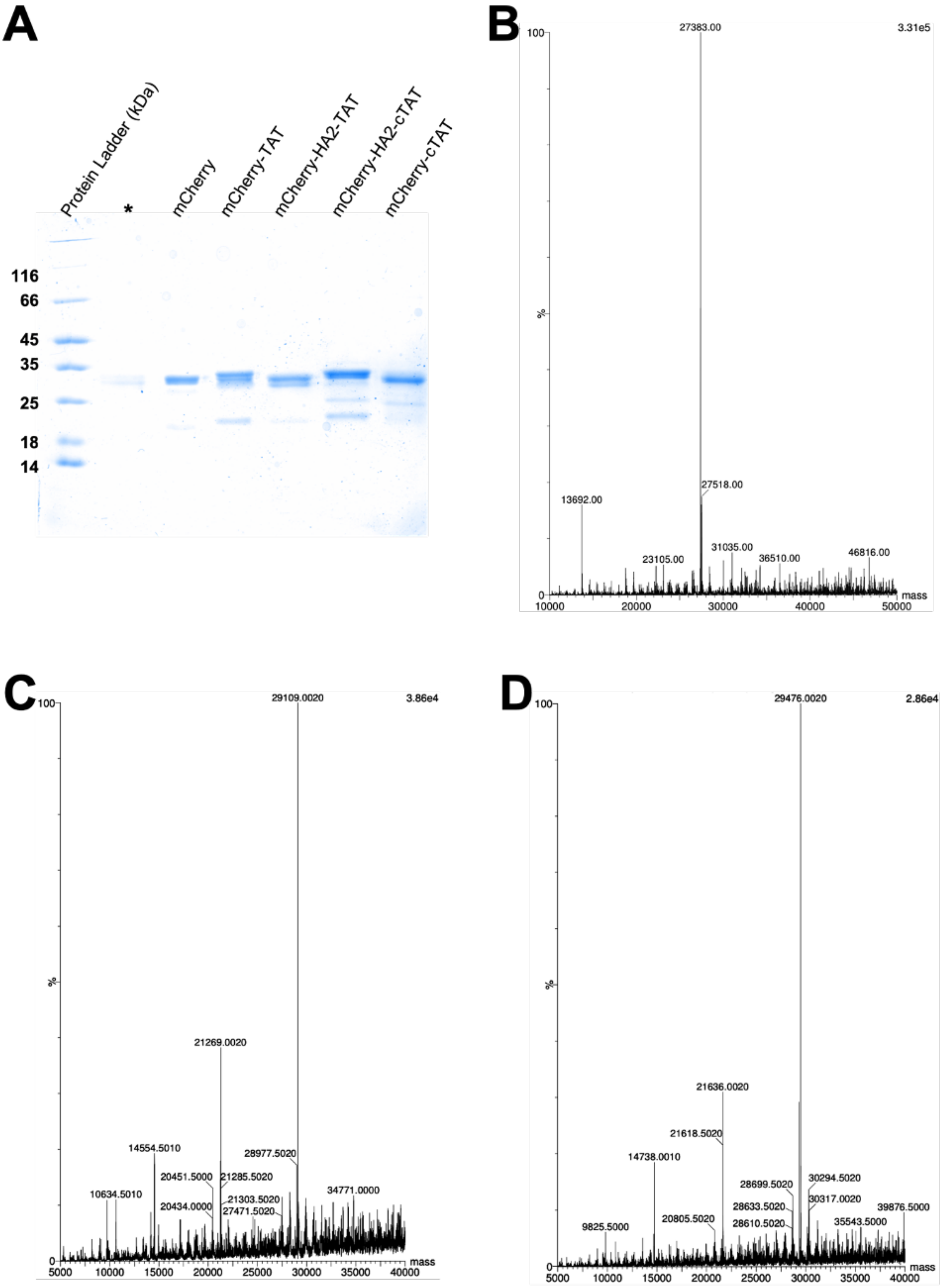

Figure S2. Characterisation of mCherry-CPP after purification. (A) SDS-PAGE of mCherry-CPP constructs after purification. The samples show secondary splicing products that could not be purified from the target protein by Ni^{2+}-affinity chromatography, cation exchange chromatography or gel filtration. Protein concentration was determined by absorbance spectroscopy at 585 nm, hence the impurities were not accounted for. * A blank lane with no protein sample loaded, faint band is due to leaking from loading mCherry lane. (B-D) Mass spectrometry (MS) of mCherry, mCherry-TAT and mCherry-cTAT. Chromophore formation leads to a loss of 20 Da from the mass calculated from the sequence in all constructs as water is released (H_2O: 18 Da), and a double bond is formed (2H: 2 Da). The His_6-tag was cleaved from mCherry-TAT and mCherry-cTAT with TEV protease during purification. This mass loss (-GHHHHHH: 897.91 Da) was subtracted from the calculated mass. (B) mCherry, calculated mass: 27516.01 Da, calculated mass (-M): 27384.82, found mass: 27383.00 Da. (C) mCherry-TAT, calculated mass: 29109.90,

found mass: 29109.0020 Da. (D) mCherry-cTAT, calculated mass (oxidised, circular): 29477.36 Da, calculated mass (reduced, linear): 29479.36 Da, found mass: 29476.0020 Da.

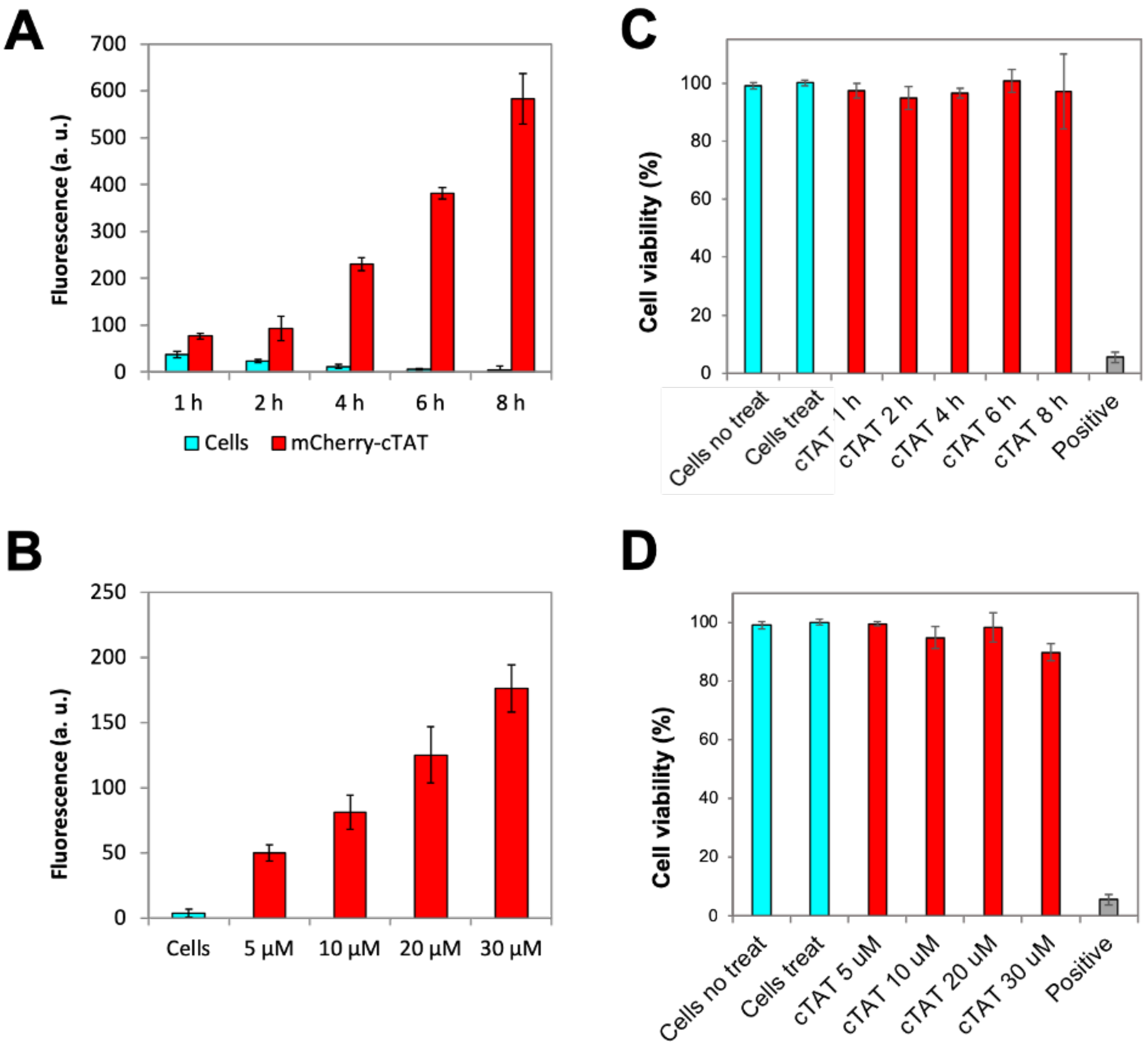

Figure S3. Incubation studies of mCherry-cTAT. (A) 250,000 cells per well of a 24 clear well-plate were incubated for 1 to 8 hours with mCherry-cTAT (10 µM) in DMEM (serum free). Cells were washed three times with heparin in PBS and once with PBS before detaching them with trypsin and resuspending them in PBS (450 µL) and analyzing by FACS. (B) 250,000 cells per in a 24 clear well-plate) were incubated for 2 hours with mCherry-cTAT in varying concentrations from 5 to 30 µM in DMEM (serum free). Cells were washed three times with heparin in PBS and once with PBS before detaching them with trypsin and resuspending them in PBS (450 µL) and analysing by FACS. (C) 20,000 cells per well in a 96 black well-plate were incubated for 1 to 8 hours with mCherry-cTAT (10 µM) in DMEM (serum free). Cells were washed three times with heparin in PBS and once with PBS before adding DMEM supplemented with 10% FBS (100 µL) and Celltiter Blue reagent (20 µL). Cells were incubated with the reagent for 4 hours and fluorescence at 610 nm was measured using a plate reader. Untreated cells were kept in DMEM (supplemented with 10% FBS) during all the experiment, and no heparin washes were performed on them. All values were normalized to non-treated cells (considered to be 100% viability). Triton X-100 (0.01%) was employed as positive control for cell death. (D) 20,000 cells per well in a 96 black well-plate) were incubated for 2 hours with mCherry-cTAT in concentrations varying from 5 to 30 µM in DMEM (serum free). Cells were washed three times with heparin in PBS and once with PBS before adding DMEM supplemented with 10% FBS (100 µL) and Celltiter Blue reagent (20 µL). Cells were incubated with the reagent for 4 hours and fluorescence at 610 nm was measured by a plate reader. Untreated cells were kept in DMEM (supplemented with 10% FBS) during all the experiment, and no heparin washes were performed on them. All values were normalized to non-treated cells (considered to be 100% viability). Triton X-100 (0.01%) was employed as positive control for cell death.

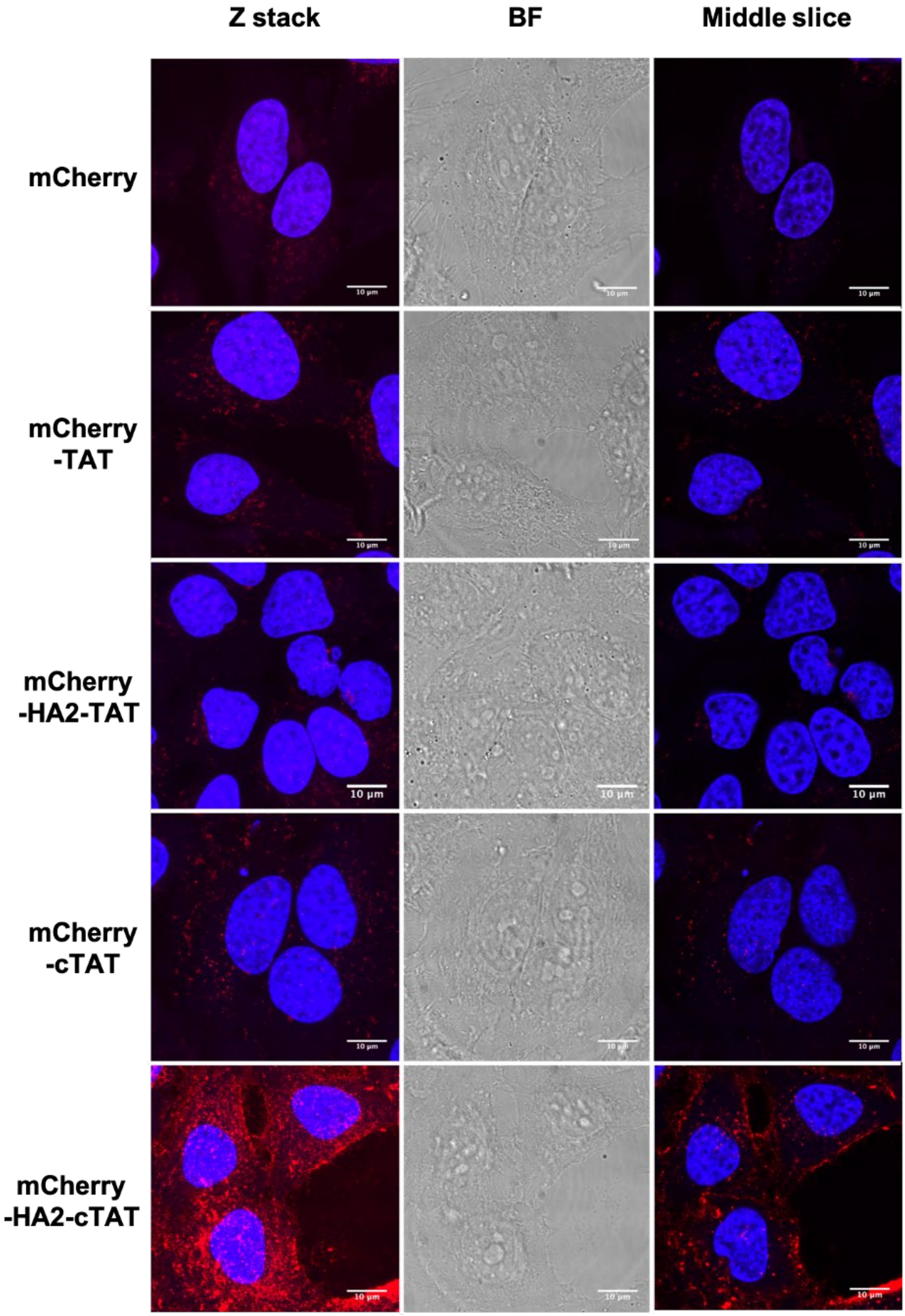

Figure S4. Localisation of mCherry-CPP proteins in HeLa cells. HeLa cells were incubated for 2 h with protein samples (10 µM) in DMEM media. Cells were washed three times with a solution of heparin in PBS to remove plasma membrane bound proteins, then in PBS to remove heparin. Samples were treated with the nuclear stain Hoechst 33342 (10 µg/ml) in DMEM media (supplemented with 10% FBS) for 5 minutes at the very end of the experiment. Nuclei are shown in blue and mCherry in red. Images were taken in the Z axis, from the bottom to the top of the cells, every 0.5 µm (n = 15 – 17). Left hand images correspond to a composite of the Z stack images selecting the brightest pixel from every layer. Middle images correspond to the bright-field (BF) images of the top of the cells. Right hand images correspond to the middle layer of the stack, i.e. the middle of the cells.

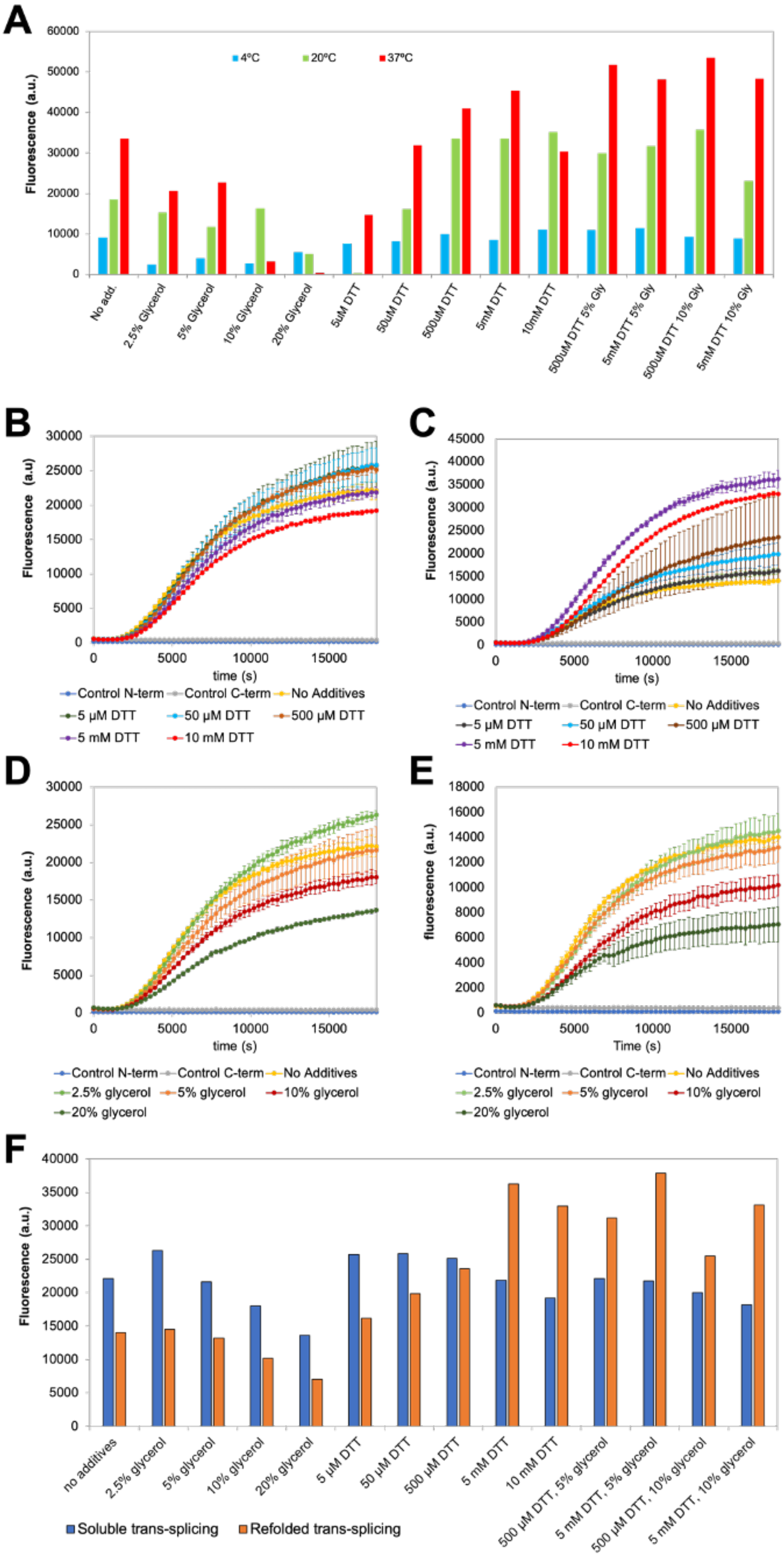

Figure S5. Kinetic characterisation of *Npu* DnaE *trans*-splicing efficiency under different conditions. N-terminal and C-terminal constructs were buffer-exchanged in splicing buffer (20 mM Tris pH 7.3, 300 mM NaCl, 2 mM EDTA) prior to the experiments, and stored as a 30 μM stock solution. Additives were added when necessary from stock solutions in splicing buffer: glycerol added from a 50% solution and DTT from a freshly prepared 500 mM stock. Final solutions of each construct at 5 μM in a final volume of 200 μl were used for all experiments. Measurements were performed in triplicate. N-terminal intein was purified from soluble fraction after sonication as well as from inclusion bodies and efficiency between soluble and refolded was compared in the experiments. C-terminal intein was purified from inclusion bodies. (A) Screening of trans-splicing with soluble N-terminal construct at 4, 20 and 37 ºC. (B) Effect of increasing concentration of DTT on soluble N-terminal trans-splicing. (C) Effect of increasing concentration of DTT in refolded N-terminal trans-splicing. (D) Effect of increasing concentration of glycerol in soluble N-terminal trans-splicing. (E) Effect of increasing concentration of glycerol in refolded N-terminal trans-splicing. (F) Comparison of soluble and refolded N-terminal splicing efficiency at 37 ºC after 5 hours. Highest activity was observed for refolded N-terminal construct with DTT and glycerol.

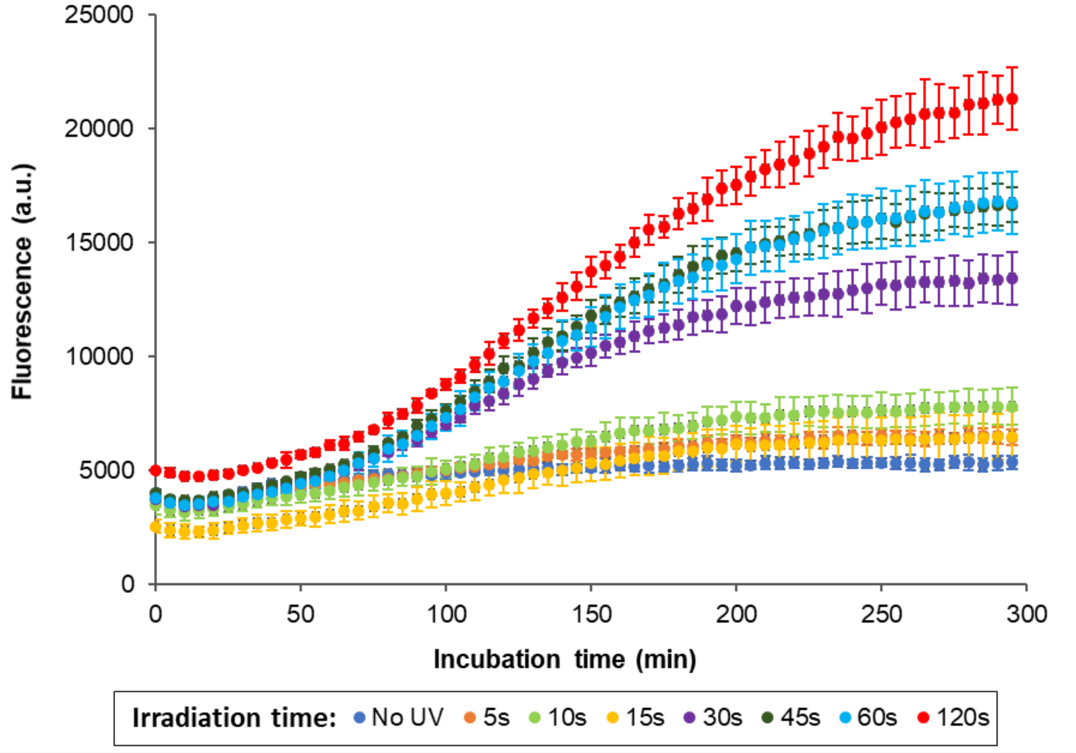

Figure S6. mCherry fluorescence increase after varying illumination times. mCherry fluorescence up to 5 hours after irradiating the sample at 365 nm for times varying from 5 to 120 seconds.

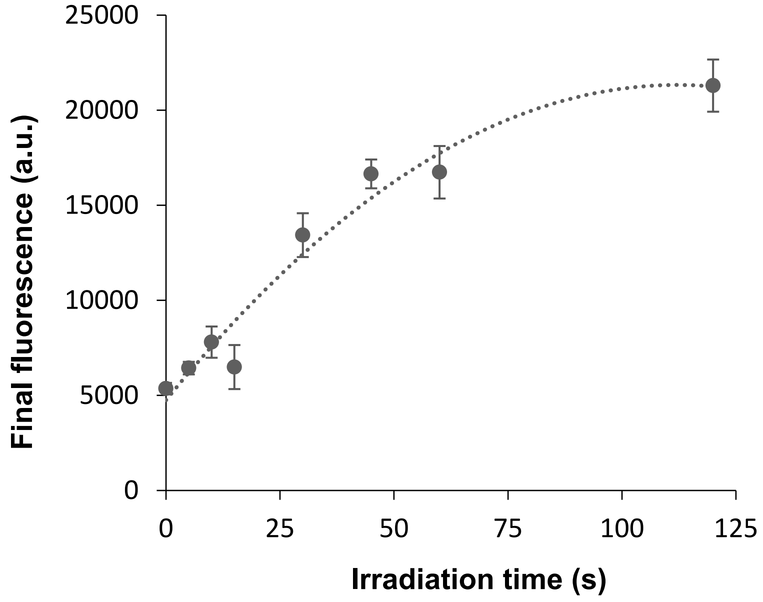

Figure S7. Final mCherry fluorescence values varying illumination times. mCherry fluorescence 5 hours after irradiating the sample at 365 nm for times varying from 5 to 120 seconds.

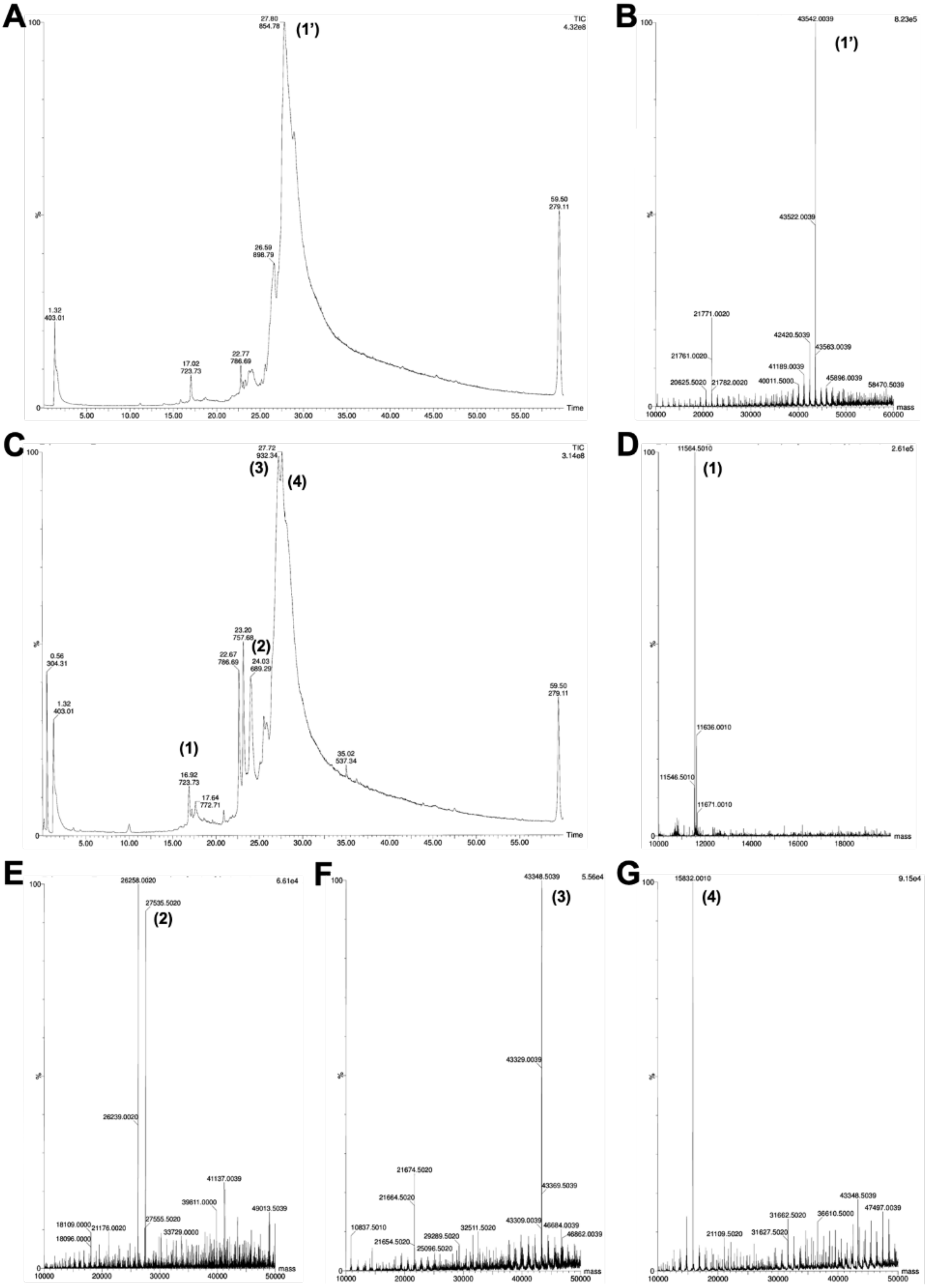

Figure S8. MS characterisation of photocaged intein-split mCherry before and after irradiation (and overnight incubation at 37 ºC). (A) Total ion current (TIC) chromatogram of a sample of photocaged protein pre-irradiation. (B) MS of main peak corresponds to photocaged full-length intein-split mCherry (retention time: 27.80 min, calculated mass: 43544.97 Da, found mass: 43542.0039 Da). (C) TIC chromatogram of 2-minute illuminated sample after overnight incubation at 37 ºC. The main peak from pre-irradiated chromatogram is split into two, and smaller peaks appeared. Peaks that could be identified are numbered 1 to 4. (D) MS corresponding to cleaved C-terminal mCherry (retention time: 16.90 min, calculated mass: 11564.94 Da, found mass: 11564.5010Da). (E) MS corresponding to non-matured mCherry (retention time: 24.03 min, calculated mass: 27536.01 Da, found mass: 27535.5020 Da). (F) MS of decaged full-length intein-split mCherry (retention time: 27.31 min,

calculated mass: 43350.97 Da, found mass: 43348.5039 Da). (G) MS of *Npu* DnaE intein (retention time: 27.67 min, calculated mass: 15832.97 Da, found mass: 15832.0010 Da).

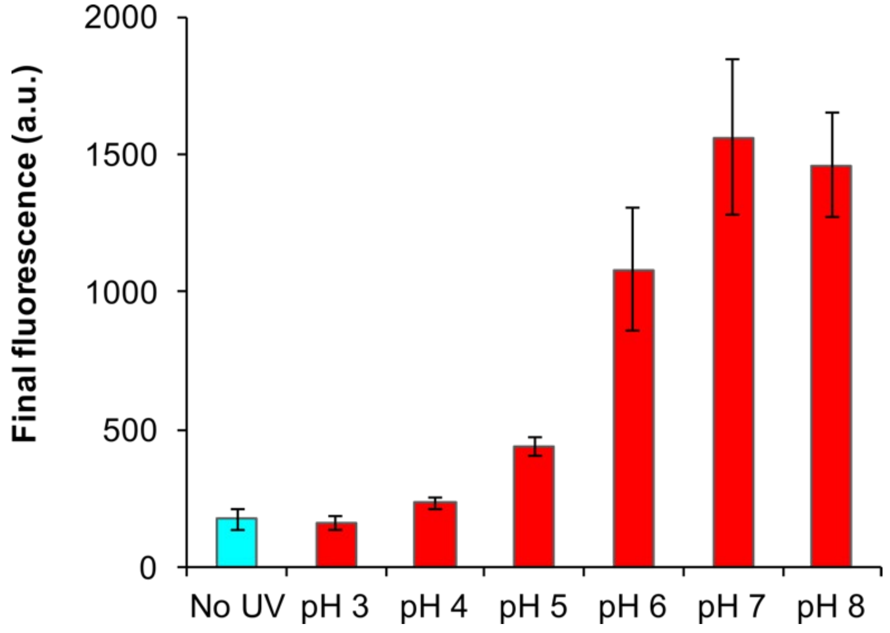

Figure S9. pH dependance of intein splicing. Fluorescence after 5 hours incubation following after 2 minutes 365 nm irradiation for *cis*- and *trans*-splicing *Npu* DnaE mCherry variants (5 µM) under the same conditions performed in triplicate.

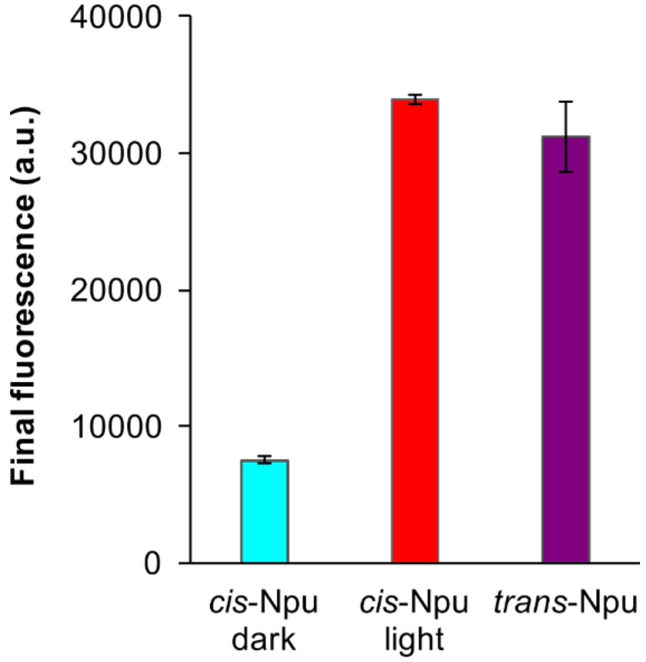

Figure S10. Characterisation of photodecaging of purified intein-interrupted mCherry. Cherry fluorescence after 5 hours incubation following 2 minutes of 365 nm irradiation (2 µM protein) in buffers at different pH values. Bars indicate the mean of triplicate experiments and error bars indicate the standard deviation.

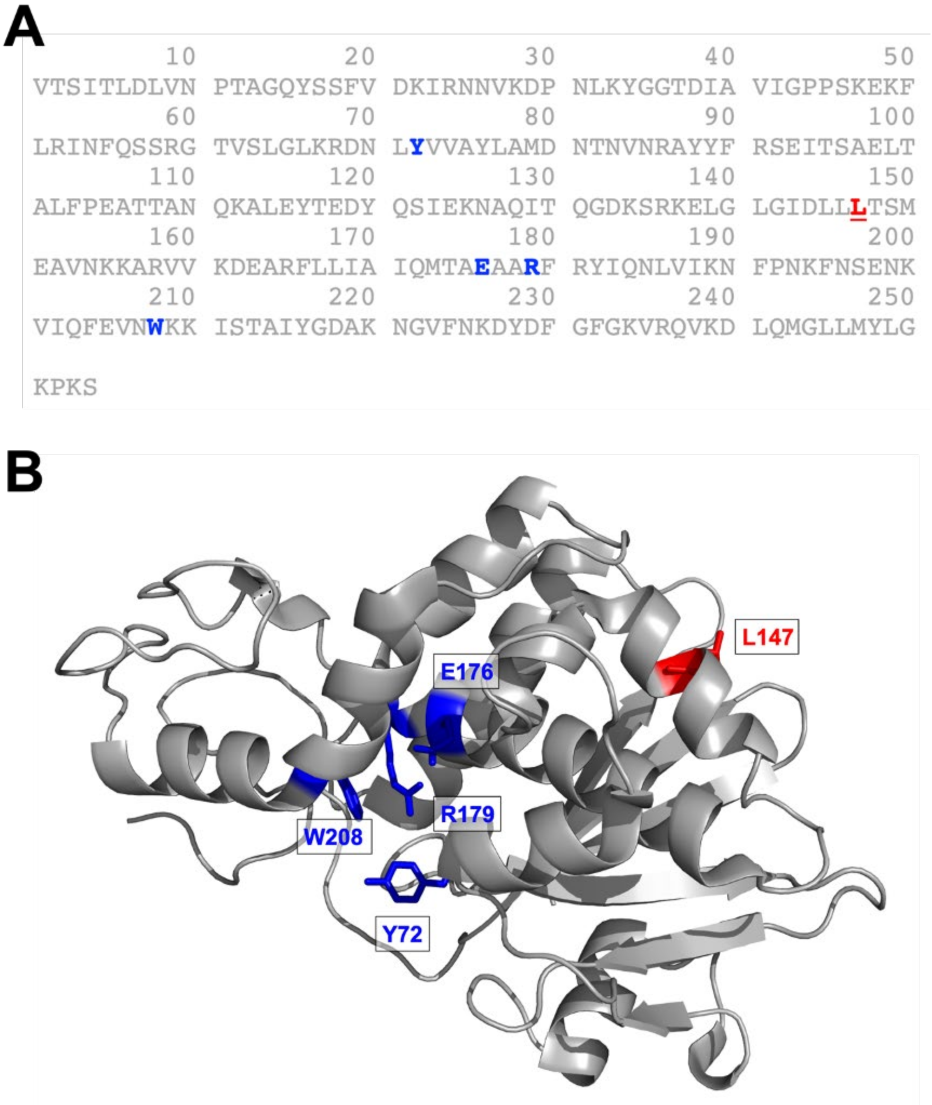

Figure S11. Sequence and structure of Saporin-S6, a ribosome-inactivating protein. Catalytic residues are shown in blue. The catalytic triad consists of E176, R179 and W208. Y72 targets the adenine of interest from the ribosomal tRNA. The location selected to locate the intein (L147, shown in red) was located in between the catalytic site and the residue binding the target adenine, and it presented desirable flanking residues for splicing. (A) Sequence of saporin-S6. (B) Crystal structure of saporin-S6 (PDB: 1QI7).**Figure S3.** Incubation studies of mCherry-cTAT. (A) 250,000 cells per well of a 24 clear well-plate were incubated for 1 to 8 hours with mCherry-cTAT (10 µM) in DMEM (serum free). Cells were washed three times with heparin in PBS and once with PBS before detaching them with trypsin and resuspending them in PBS (450 µL) and analyzing by FACS. (B) 250,000 cells per in a 24 clear well-plate) were incubated for 2 hours with mCherry-cTAT in varying concentrations from 5 to 30 µM in DMEM (serum free). Cells were washed three times with heparin in PBS and once with PBS before detaching them with trypsin and resuspending them in PBS (450 µL) and analysing by FACS. (C) 20,000 cells per well in a 96 black well-plate were incubated for 1 to 8 hours with mCherry-cTAT (10 µM) in DMEM (serum free). Cells were washed three times with heparin in PBS and once with PBS before adding DMEM supplemented with 10% FBS (100 µL) and Celltiter Blue reagent (20 µL). Cells were incubated with the reagent for 4 hours and fluorescence at 610 nm was measured using a plate reader. Untreated cells were kept in DMEM (supplemented with 10% FBS) during all the experiment, and no heparin washes were performed on them. (D) 20,000 cells per well in a 96 black well-plate) were incubated for 2 hours with mCherry-cTAT in concentrations varying from 5 to 30 µM in DMEM (serum free). Cells were washed three times with heparin in PBS and once with PBS before adding DMEM supplemented with 10% FBS (100 µL) and Celltiter Blue reagent (20 µL). Cells were incubated with the reagent for 4 hours and fluorescence at 610 nm was measured by a plate reader. Untreated cells were kept in DMEM (supplemented with 10% FBS) during all the experiment, and no heparin washes were performed on them.

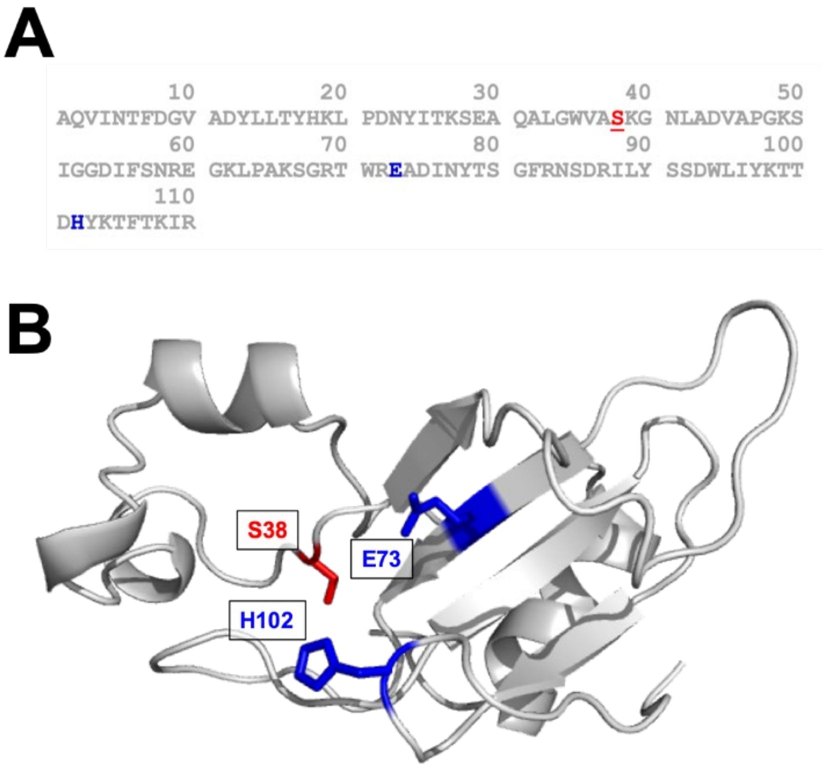

Figure S12. Sequence and structure of barnase, a bacterial ribonuclease. Catalytic residues are shown in blue. The catalytic dyad consists of E73 and H102. The location selected to locate the intein (S38, shown in red) was located in between the catalytic site and the residue binding the target adenine, and it presented desirable flanking residues for splicing. (A) Sequence of barnase. (B) Crystal structure of barnase (PDB: 1A2P).

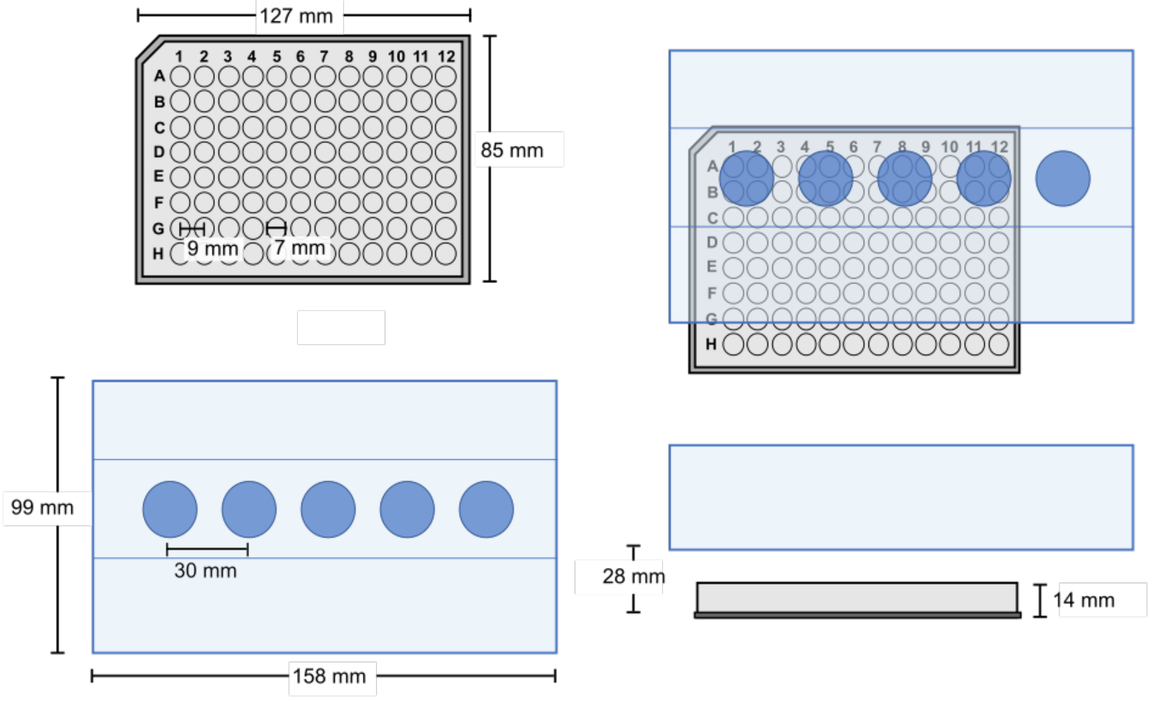

Figure S13. Irradiation set-up to trigger intein splicing and saporin activation in HeLa cells. 96-well plate with HeLa cells depicted in black and LED lamp (5 UV-LED strip unit, UV Light Technology Limited) depicted in blue.

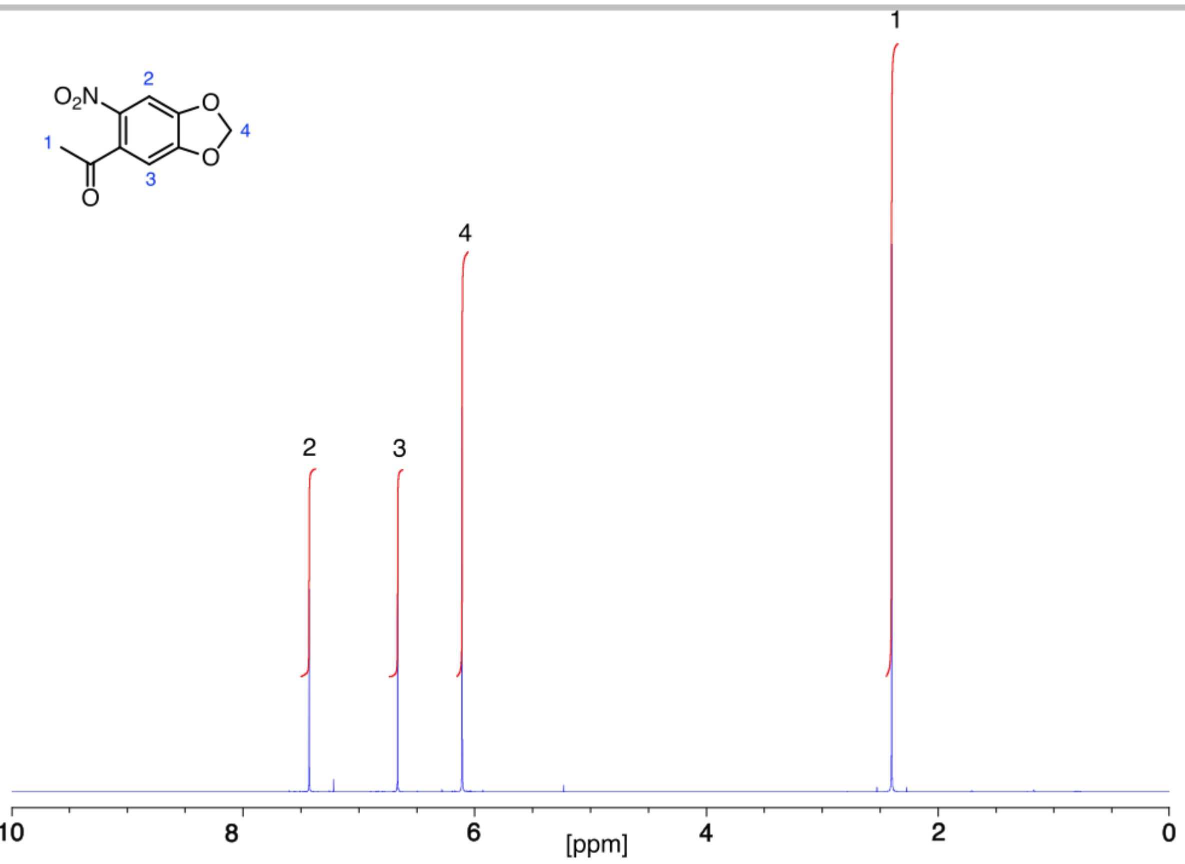

Figure S14. ^{1}H-NMR (500 MHz, $CDCl_3$) of 3’,4’-(methylendioxy)-6’-nitroacetophenone (2).

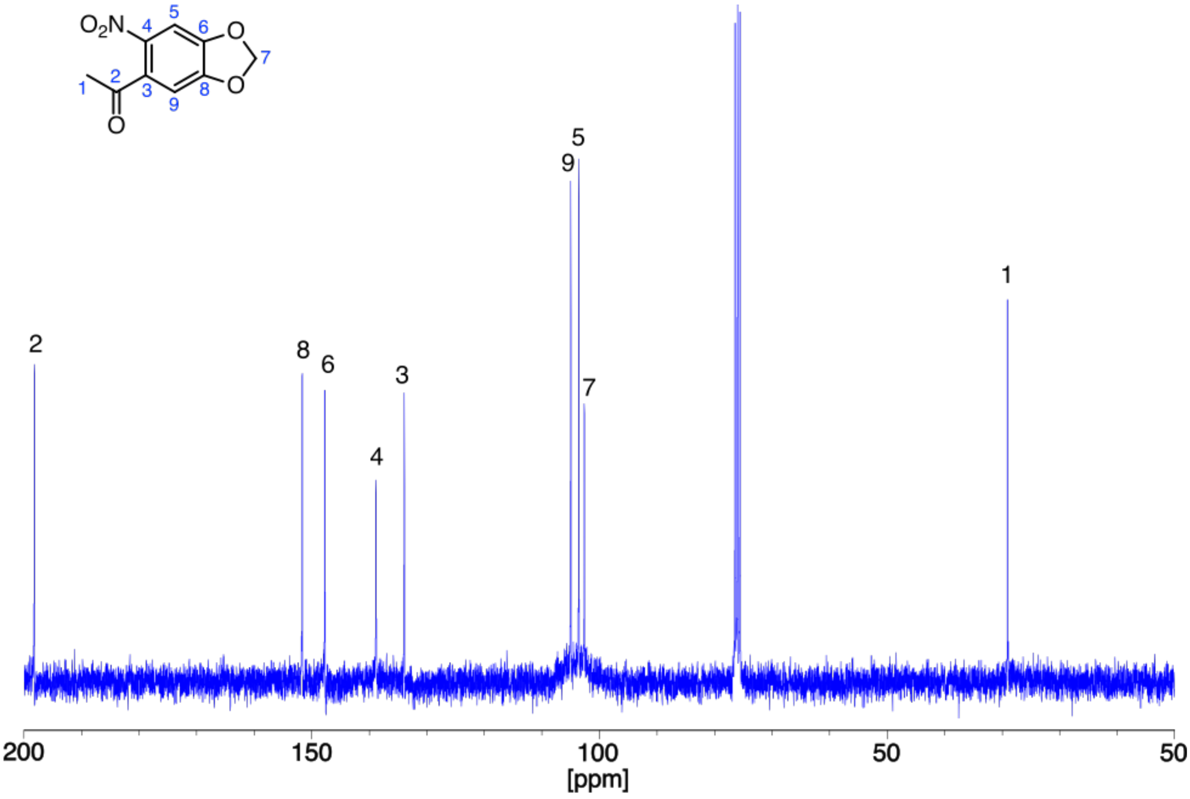

Figure S15. ^{13}C-NMR (75 MHz, $CDCl_3$) of 3’,4’-(methylendioxy)-6’-nitroacetophenone (2).

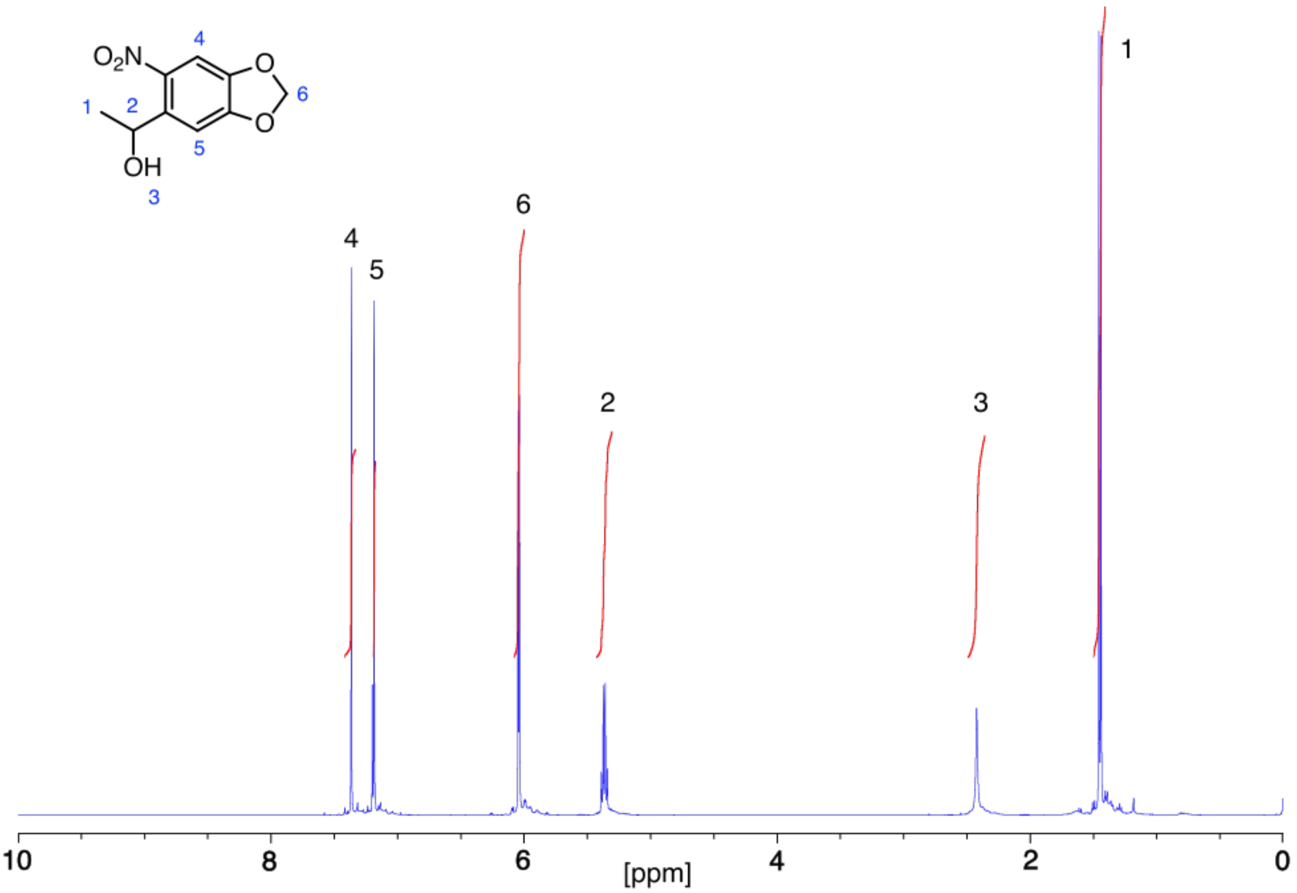

Figure S16. ^{1}H-NMR (400 MHz, $CDCl_3$) of methylnitropiperonyl alcohol (3).

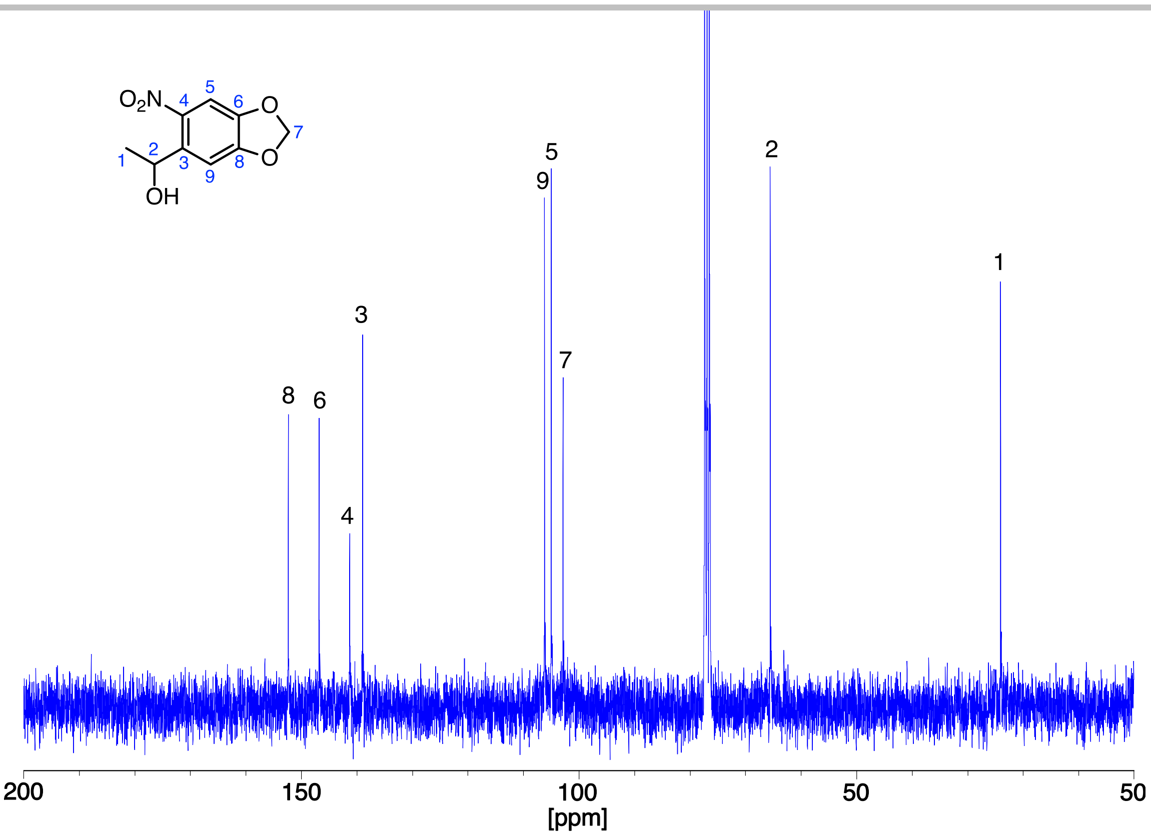

Figure S17. ^{13}C-NMR (75 MHz, $CDCl_3$) of methylnitropiperonyl alcohol (3).

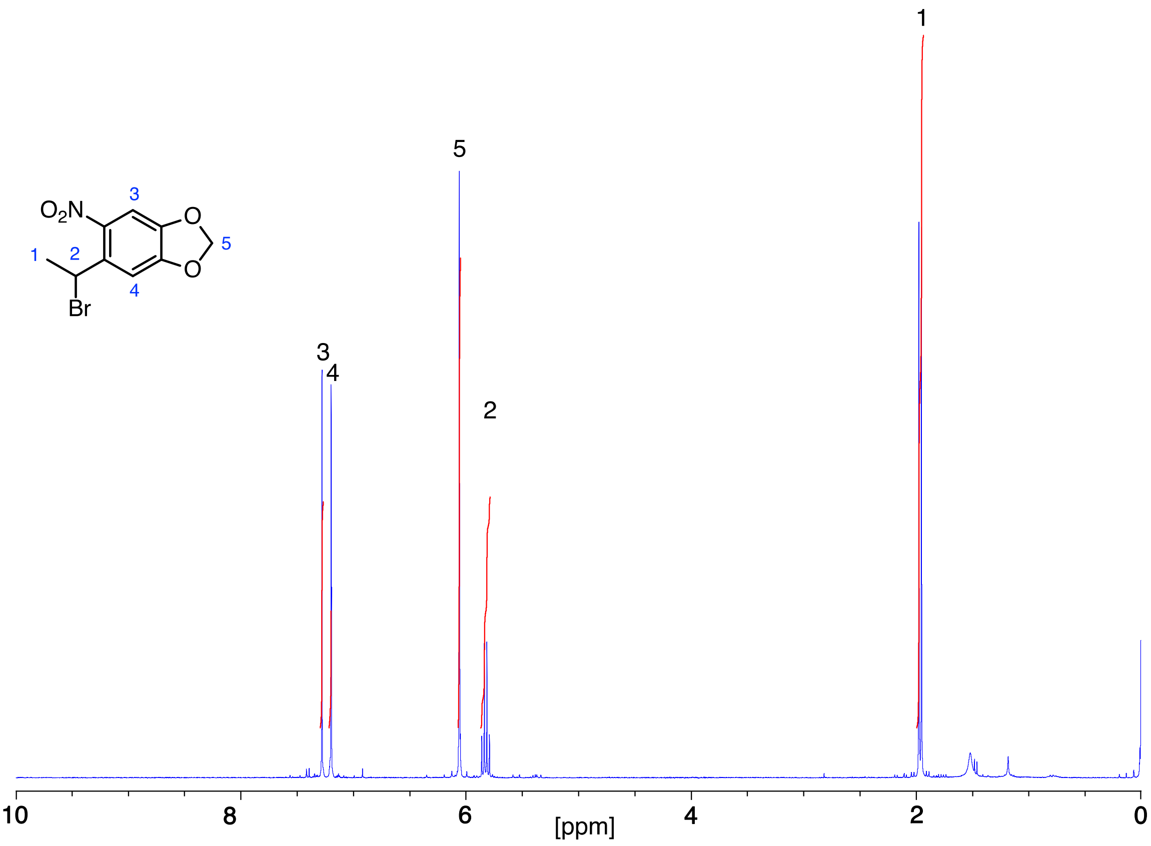

Figure S18. ^{1}H-NMR (300 MHz, $CDCl_3$) of (*R*,*S*)-1-Bromo-1-[4',5'-(methylendioxy)-2'-nitrophenyl]ethane (4).

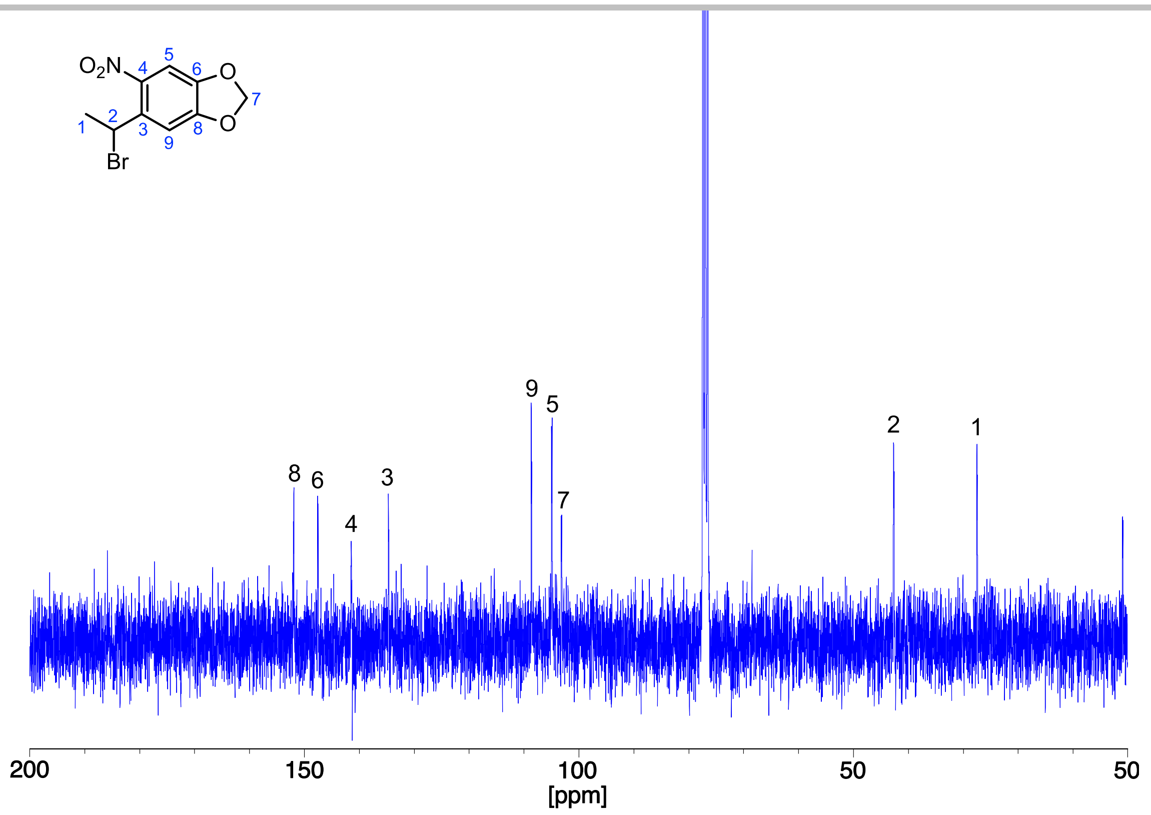

Figure S19. ^{13}C-NMR (75 MHz, $CDCl_3$) of (*R*,*S*)-1-Bromo-1-[4',5'-(methylendioxy)-2'-nitrophenyl]ethane (4).

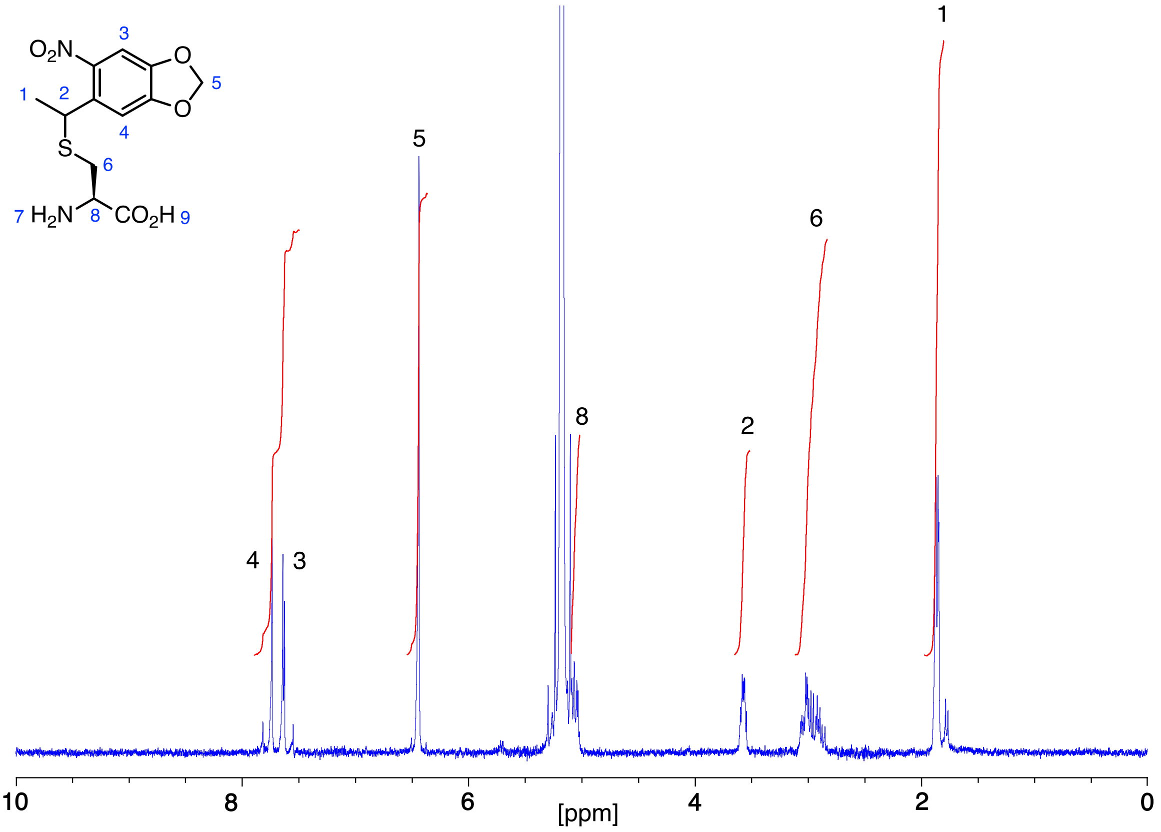

Figure S20. ^{1}H-NMR (400 MHz, NaOD/D_2O) of *S*-(1-(6-nitrobenzo[d][1,3]dioxol-5-yl)ethyl)-*L*-cysteine (1).

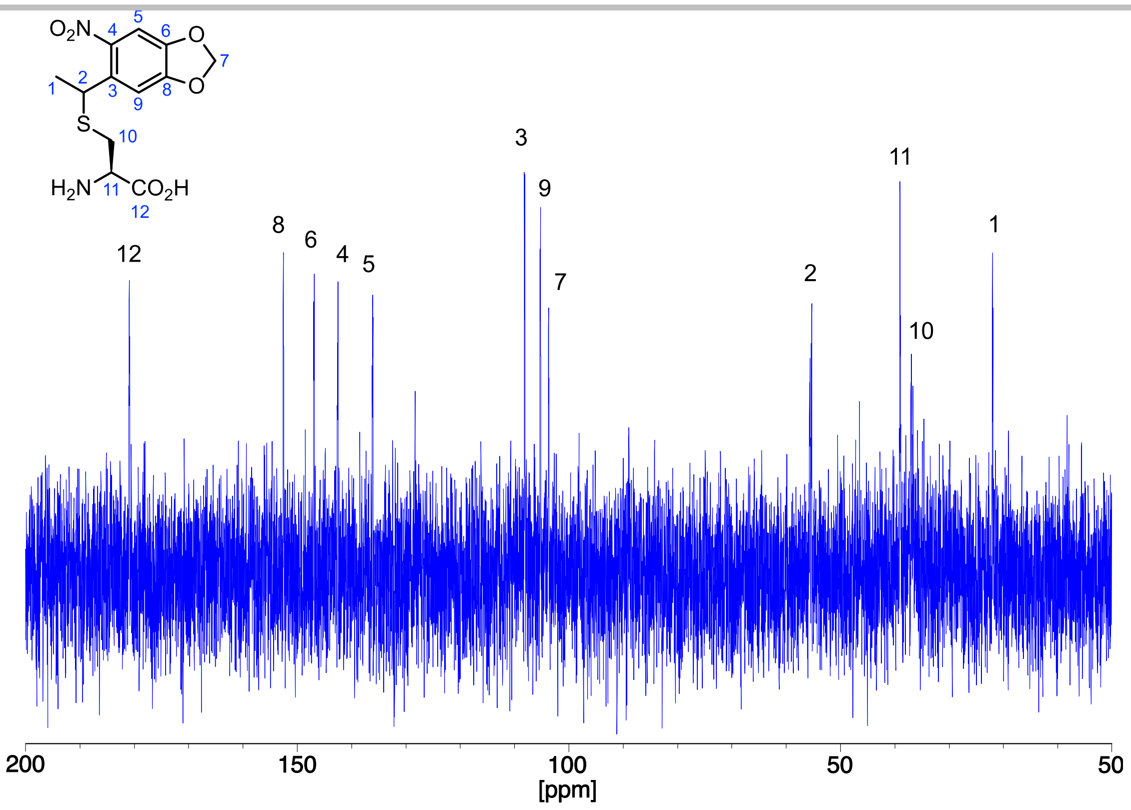

Figure S21. ^{13}C-NMR (125 MHz, $CDCl_3$) of *S*-(1-(6-nitrobenzo[d][1,3]dioxol-5-yl)ethyl)-*L*-cysteine (1).

Table S1. Gene sequences. Amber codon shown in red, sequence encoding intein shown in blue, *Bsa*I recognisition sites shown in grey.

Plasmid	Source	Cloning sites	Gene sequence
mCh-(144TAG)-int-erry/ pET30b(+)	Purchased from AddGene	*Nde*I-*Xho*I	CATATGGTTAGCAAAGGCGAGGAAGACAATATGGCGATCATCAAGGAGTTTATGCGTTTC<br>AAGGTTCACATGGAGGGCAGCGTTAATGGCCACGAGTTCGAAATCGAGGGTGAAGGTGA<br>AGGTCGTCCGTACGAGGGTACCCAGACCGCGAAGCTGAAAGTTACCAAGGGTGGCCCG<br>CTGCCGTTTGCGTGGGATATCCTGAGCCCGCAATTTATGTACGGCAGCAAGGCGTATGTT<br>AAACACCCGGCGGACATTCCGGATTACCTGAAACTGAGCTTCCCGGAGGGTTTTAACTG<br>GGAACGTGTGATGAACTTTGAGGATGGTGGCGTGGTTACCGTTACCCAGGACAGCAGCC<br>TGCAAGATGGTGAATTCATCTATAAGGTGAAACTGCGTGGCACCAACTTTCCGAGCGACG<br>GTCCGGTTATGCAATAGCTGAGCTACGAAACCGAGATTCTGACCGTGGAATATGGCCTG<br>CTGCCGATCGGCAAGATTGTTGAGAAACGTATCGAATGCACCGTGTACAGCGTTGACAAC<br>AACGGCAACATTTATACCCAGCCGGTGGCGCAATGGCACGATCGTGGCGAACAAGAGGT<br>TTTCGAGTACTGCCTGGAAGACGGTAGCCTGATCCGTGCGACCAAGGACCACAAATTCAT<br>GACCGTGGATGGTCAGATGCTGCCGATCGACGAGATTTTTGAACGTGAGCTGGACCTGA<br>TGCGTGTTGATAACCTGCCGAACATCAAGATTGCGACCCGTAAGTACCTGGGCAAACAGA<br>ACGTGTATGACATCGGTGTTGAACGTGATCACAACTTCGCGCTGAAAAACGGCTTTATTG<br>CGAGCAACTGCATGACCATGGGTTGGGAGGCGAGCACCGAACGTATGTACCCGGAGGA<br>TGGCGCGCTGAAGGGTGAAATCAAACAGCGTCTGAAGCTGAAAGACGGTGGCCACTACG<br>ATGCGGAAGTGAAGACCACCTATAAAGCGAAGAAACCGGTGCAACTGCCGGGTGCGTAT<br>AACGTTGACATCAAGCTGGATATTCTGAGCCACAACGAAGACTACACCATTGTTGAACAG<br>TATGAGCGTGCGGAAGGTCGCCATAGCACCGGCGGCATGGACGAACTGTATAAGCATCA<br>TCATCATCATCACTGACTCGAG
PCC2RS/ pUC	Obtained from Dr. Yu-Hsuan Tsai	N/A	ATGATGGATAAAAAACCGCTGGATGTGCTGATTAGCGCGACCGGCCTGTGGATGAGCCG<br>TACCGGCACCCTGCATAAAATCAAACATCATGAAGTGAGCCGCAGCAAAATCTATATTGA<br>AATGGCGTGCGGCGATCATCTGGTGGTGAACAACAGCCGTAGCTGCCGTACCGCGCGT<br>GCGTTTCGTCATCATAAATACCGCAAAACCTGCAAACGTTGCCGTGTGAGCGATGAAGAT<br>ATCAACAACTTTCTGACCCGTAGCACCGAAAGCAAAAACAGCGTGAAAGTGCGTGTGGTG<br>AGCGCGCCGAAAGTGAAAAAAGCGATGCCGAAAAGCGTGAGCCGTGCGCCGAAACCGC<br>TGGAAAATAGCGTGAGCGCGAAAGCGAGCACCAACACCAGCCGTAGCGTTCCGAGCCC<br>GGCGAAAAGCACCCCGAACAGCAGCGTTCCGGCGTCTGCGCCGGCACCGAGCCTGACC<br>CGCAGCCAGCTGGATCGTGTGGAAGCGCTGCTGTCTCCGGAAGATAAAATTAGCCTGAA

			CATGGCGAAACCGTTTCGTGAACTGGAACCGGAACTGGTGACCCGTCGTAAAAACGATTT<br>TCAGCGCCTGTATACCAACGATCGTGAAGATTATCTGGGCAAACTGGAACGTGATATCAC<br>CAAATTTTTTGTGGATCGCGGCTTTCTGGAAATTAAAAGCCCGATTCTGATTCCGGCGGA<br>ATATGTGGAACGTATGGGCATTAACAACGACACCGAACTGAGCAAACAAATTTTCCGCGT<br>GGATAAAAACCTGTGCCTGCGTCCGATGCTGGCCCCGACCCTGTATAACTATCTGCGTAA<br>ACTGGATCGTATTCTGCCGGGTCCGATCAAAATTTTTGAAGTGGGCCCGTGCTATCGCAA<br>AGAAAGCGATGGCAAAGAACACCTGGAAGAATTCACCATGGTTCAGTTTGCGCAAATGG<br>GCAGCGGCTGCACCCGTGAAAACCTGGAAGCGCTGATCAAGGAATTCCTGGATTATCTG<br>GAAATCGACTTCGAAATTGTGGGCGATAGCTGCATGGTGTATGGCGATACCCTGGATATT<br>ATGCATGGCGATCTGGAACTGAGCAGCGCGATGGTGGGTCCGGTTAGCCTGGATCGTGA<br>ATGGGGCATTGATAAACCGTGGATTGGCGCGGGGTTTGGCCTGGAACGTCTGCTGAAAG<br>TGATGCATGGCTTCAAAAACATTAAACGTGCGAGCCGTAGCGAAAGCTACTATAACGGCA<br>TTAGCACGAACCTG
Sap-(148TAG)-int-orin	Purchased from GeneArt Thermofisher Scientific	*BsaI-BsaI*	CATTGGTCTCTTATGGTTACCAGCATTACCCTGGATCTGGTTAATCCGACCGCAGGTCAG<br>TATAGCAGCTTTGTTGATAAAATTCGCAACAACGTGAAAGACCCGAATCTGAAATATGGTG<br>GCACCGATATTGCAGTTATTGGTCCGCCTAGCAAAGAAAAATTTCTGCGCATTAACTTTCA<br>GAGCAGCCGTGGCACCGTTAGCCTGGGTCTGAAACGTGATAATCTGTATGTTGTTGCATA<br>TCTGGCCATGGATAATACCAATGTTAATCGTGCCTATTATTTCCGCAGCGAAATTACCAGC<br>GCAGAACTGACCGCACTGTTTCCGGAAGCAACCACCGCAAATCAGAAAGCACTGGAATA<br>TACCGAAGATTATCAGAGCATTGAAAAGAACGCACAGATTACCCAGGGTGATCAGAGCCG<br>TAAAGAATTAGGTCTGGGTATTGATCTGCTGTGACTGAGCTATGAAACCGAAATTCTGAC<br>CGTTGAATATGGTCTGCTGCCGATTGGTAAAATTGTGGAAAAACGTATTGAGTGCACCGT<br>GTATAGCGTGGATAATAACGGTAACATTTATACCCAGCCGGTTGCACAGTGGCATGATCG<br>TGGTGAACAAGAAGTTTTTGAATACTGTCTGGAAGATGGTAGCCTGATTCGTGCAACCAA<br>AGATCACAAATTTATGACCGTGGATGGTCAGATGCTGCCCATTGATGAAATTTTTGAACGT<br>GAACTGGATCTGATGCGTGTGGATAATCTGCCGAATATCAAAATTGCGACCCGTAAATAT<br>CTGGGCAAACAGAACGTTTATGATATTGGTGTGGAACGCGATCATAACTTTGCACTGAAA<br>AATGGTTTTATCGCCAGCAATTGTACCAGCATGGAAGCCGTTAACAAAAAAGCACGTGTT<br>GTTAAAGATGAAGCCCGTTTTCTGCTGATTGCAATTCAGATGACCGCAGAAGCAGCACGT<br>TTTCGTTATATTCAGAATCTGGTGATCAAGAACTTCCCGAACAAATTCAACAGCGAGAACA<br>AAGTGATTCAGTTTGAGGTCAACTGGAAAAAAATCAGCACCGCCATTTATGGTGATGCCA<br>AAAATGGTGTGTTCAACAAAGATTATGATTTTGGCTTTGGTAAAGTGCGCCAGGTTAAAGA<br>TCTGCAAATGGGTCTGCTGATGTATCTGGGTAAACCGAAAGAGAAGAGACCTTAC
Barn-(39TAG)-int-ase	Purchased from GeneArt Thermofisher Scientific	*BsaI-BsaI*	CATTGGTCTCTTATGGCACAGGTGATTAATACCTTTGATGGTGTTGCCGATTATCTGCAGA<br>CCTATCATAAACTGCCGGATAACTATATCACCAAAAGCGAAGCACAGGCATTAGGTTGGG<br>TTGCATAGCTGAGCTATGAAACCGAAATTCTGACCGTTGAATATGGTCTGCTGCCGATTG<br>GTAAAATTGTGGAAAAACGTATTGAGTGCACCGTGTATAGCGTGGATAATAACGGTAACA<br>TTTATACCCAGCCGGTTGCACAGTGGCATGATCGTGGTGAACAAGAAGTTTTTGAATACT<br>GTCTGGAAGATGGTAGCCTGATTCGTGCAACCAAAGATCACAAATTTATGACCGTGGATG<br>GTCAGATGCTGCCCATTGATGAAATTTTTGAACGTGAACTGGATCTGATGCGTGTTGATAA<br>TCTGCCGAATATCAAAATTGCGACCCGTAAATATCTGGGCAAACAGAACGTGTATGATATT<br>GGTGTGGAACGCGATCATAATTTCGCACTGAAAAATGGTTTTATCGCCAGCAATTGCAAA<br>GGTAATCTGGCAGATGTTGCACCGGGTAAAAGCATTGGTGGTGATATCTTTAGCAATCGC<br>GAAGGTAAACTGCCTGGTAAAAGCGGTCGTACCTGGCGTGAAGCAGATATCAATTATACC<br>AGCGGTTTTCGTAATAGCGATCGCATTCTGTATAGCAGCGATTGGCTGATCTATAAAACC<br>ACCGATCATTATCAGACCTTCACCAAAATTCGCGAGAAGAGACCTTAC

Table S2. Primers used to generate plasmids.

Primers	Section	Direction	Sequence (5' to 3')
TAG144C **SDM**[a]	N/A	FWD[b]	ATCTCGGTTTCGTAGCTCAGGCATTGCATAACCGGACCGTCGCTC
		REV[c]	CTGAGCTACGAA ACCGAGATTCTGAC
TAT-TEV **Golden Gate assembly**	Insert	FWD	CATGGGGTCTCATATGGTTAGCAAAGGCGAGGAAGACAATATGG
		REV	AGTATGGGTCTCGTCTCCTTATACAGTTCGTCCATGC
	Backbone	FWD	CGAGACCCATGGGGTCTCAGAGAATGGTCGTAAAAAACGTCGTCAGC
		REV	GAGACCCCATGGGTCTCGCATATGTATATCTCCTTCTTAAAGTTAAAC
cTAT-TEV **Golden Gate assembly**	Insert	FWD	CATGGGGTCTCATATGGTTAGCAAAGGCGAGGAAGACAATATGG
		REV	AGTATGGGTCTCGTCTCCTTATACAGTTCGTCCATGC
	Backbone	FWD	1st GACCCATGGGGTCTCAGAGAATTGCTACGGTCGTAAAAAACGTCGTCAGCG TCG<br>2nd CGGTCGTAAAAAACGTCGTCAGCGTCGTCGTTGCGAGAATCTTTATTTTCA GGG
		REV	GAGACCCCATGGGTCTCGCATATGTATATCTCCTTCTTAAAGTTAAAC
HA2-TAT-TEV **Gibson assembly**	Insert	FWD	CCACCTTGGTCTCGAGGGTGATATTATGGGTGAATGGGGTAATG
		REV	CCACCTTGGTCTCGTCACGACGACGCTGACGACGTTTTTT
	Backbone	FWD	CACCTTGGTCTCGGTGAGAATCTTTATTTTCAGGGCCACCACC
		REV	CCACCTTGGTCTCGCCCTTATACAGTTCGTCCATGCCGC
HA2-cTAT-TEV **Golden Gate assembly**	Insert	FWD	CGTTTGGGTCTCCATTGGTTAGCAAAGGCGAGGAAGAC
		REV	GCCGGTTTTCTGGGTTACTGCGAGACCCGAACA
	Backbone	FWD	TGTTCGGGTCTCGACTGCTACGGTCGTAAAAAAC
		REV	ATAATTTTGTTTAACTTTAAGAAGGAGATATACATATGATCTGGAGACCCAAACG
N-terminal mCh-DnaE **Golden Gate assembly**	Insert	FWD	CCATATTGTCTTCCTCGCCTTTGCTAACCATGTGGAGACCGATAGGC
		REV	GCCTATCGGTCTCCTCAGTTCGGCAGGTTATCAACACGCATC
	Backbone	FWD	CCTTTCGGGCTTTGTTAGCAGCCGGATCTCAGGGAGACCGATAGGC
		REV	GCCTATCGGTCTCCATGTGGTGGTGGTGGTGGTGCATATGTATATCTCC
C-terminal DnaE-erry **Golden Gate assembly**	Insert	FWD	GTTGTGGGGTCTCCCCATCAAGATTGCGACCCGTAAGTACC
		REV	GCACCGGCGGCATGGACGAACTGTATAAG
	Backbone	FWD	GTTGTGGGGTCTCGAGTAAGCGGCCGCACTCGAG
		REV	CTGAGAATCTTTATTTTCAGGGCGCCATCGAGACCCCACAAC
3xPCC**2**RS **Golden Gate assembly**	Insert 1	FWD	CTCGCAAGGTCTCGCTCGGGAGGATCCTCGGGAGTTGTC
		REV	CTCGCAAGGTCTCGTGCGG CGTGAACGCCTTA
	Insert 2	FWD	CTCGCAAGGTCTCGCGCATCCGGATCCTCGGGGAGTTGTC
		REV	CTCGCAAGGTCTCGAGGATCCGGATGCGGGCGTGAACGCC
	Insert 3	FWD	CTCGCAAGGTCTCGTCCT CGGGAGTTGTCAGCCTGT
		REV	CTCGCAAGGTCTCGCAGCGGATGCGGCGTGAACGCC
	Backbone	FWD	CTCGCAAGGTCTCGGCTGCCGGCACCTGTCCT
		REV	CTCGCAAGGTCTCGCGAGGATCCTG GCTGTGG

[a] Site-directed mutagenesis. [b] Forward, [c] Reverse.

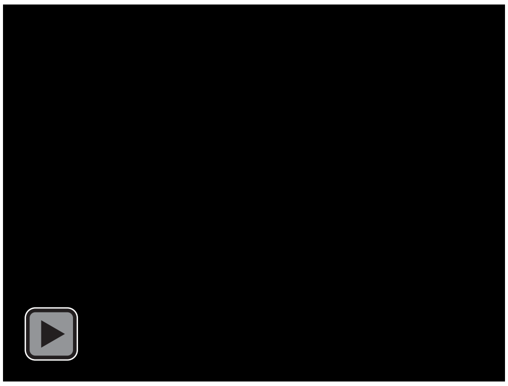

Movie S1. HeLa cells treated with mCherry. HeLa cells were incubated with mCherry (10 µM) in DMEM (serum free) for 2 hours at 37 ºC. Cells were then washed three times with heparin (0.5 mg/mL) in PBS buffer and once with PBS buffer. Cells were incubated with DMEM supplemented with 10% FBS and Hoechst 33342 (10 µg/ml) for 5 minutes before imaging. Images of the middle of the cell every 5 seconds for 3 minutes to produce the video. Cell nuclei are shown in blue and mCherry is shown in red.

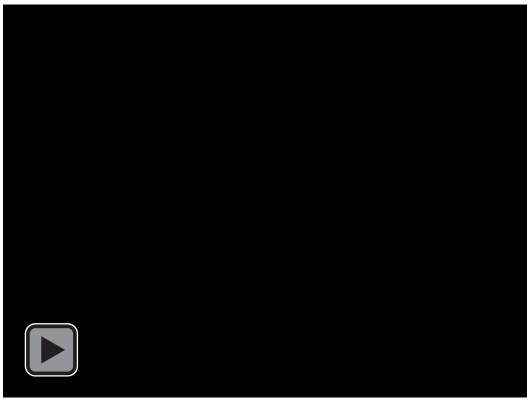

Movie S2. HeLa cells treated with mCherry-TAT. HeLa cells were incubated with mCherry-TAT (10 µM) in DMEM (serum free) for 2 hours at 37 ºC. Cells were then washed three times with heparin (0.5 mg/mL) in PBS buffer and once with PBS buffer. Cells were incubated with DMEM supplemented with 10% FBS and Hoechst 33342 (10 µg/ml) for 5 minutes before imaging. Images of the middle of the cell every 5 seconds for 3 minutes to produce the video. Cell nuclei are shown in blue and mCherry is shown in red.

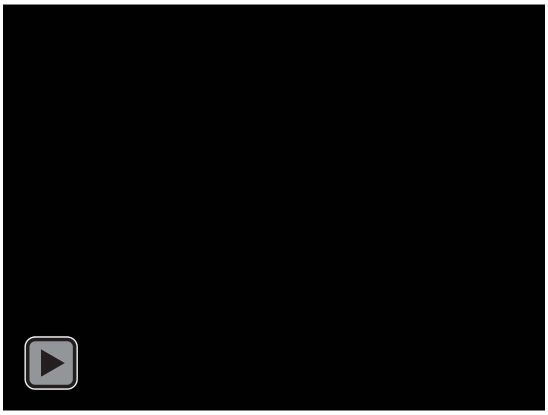

Movie S3. HeLa cells treated with mCherry-HA2-TAT. HeLa cells were incubated with mCherry-HA2-TAT (10 µM) in DMEM (serum free) for 2 hours at 37 ºC. Cells were then washed three times with heparin (0.5 mg/mL) in PBS buffer and once with PBS buffer. Cells were incubated with DMEM supplemented with 10% FBS and Hoechst 33342 (10 µg/ml) for 5 minutes before imaging. Images of the middle of the cell every 5 seconds for 3 minutes to produce the video. Cell nuclei are shown in blue and mCherry is shown in red.

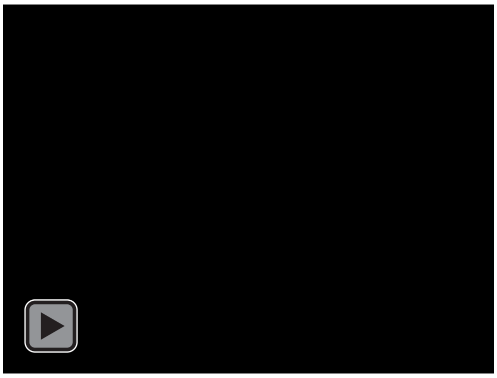

Movie S4. HeLa cells treated with mCherry-cTAT. HeLa cells were incubated with mCherry-cTAT (10 µM) in DMEM (serum free) for 2 hours at 37 ºC. Cells were then washed three times with heparin (0.5 mg/mL) in PBS buffer and once with PBS buffer. Cells were incubated with DMEM supplemented with 10% FBS and Hoechst 33342 (10 µg/ml) for 5 minutes before imaging. Images of the middle of the cell every 5 seconds for 3 minutes to produce the video. Cell nuclei are shown in blue and mCherry is shown in red.

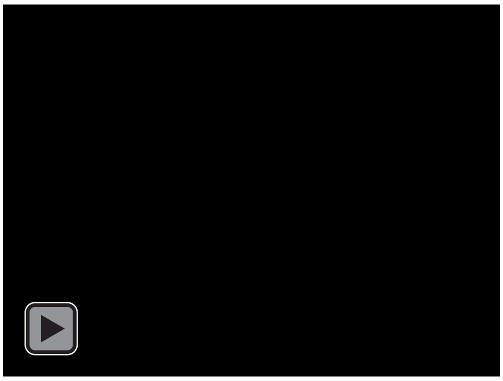

Movie S5. HeLa cells treated with mCherry-HA2-cTAT. HeLa cells were incubated with mCherry-HA2-cTAT (10 µM) in DMEM (serum free) for 2 hours at 37 ºC. Cells were then washed three times with heparin (0.5 mg/mL) in PBS buffer and once with PBS buffer. Cells were incubated with DMEM supplemented with 10% FBS and Hoechst 33342 (10 µg/ml) for 5 minutes before imaging. Images of the middle of the cell every 5 seconds for 3 minutes to produce the video. Cell nuclei are shown in blue and mCherry is shown in red.
